# Cephalosporins Interfere With Quorum Sensing and Improve the Ability of *Caenorhabditis elegans* to Survive *Pseudomonas aeruginosa* Infection

**DOI:** 10.3389/fmicb.2021.598498

**Published:** 2021-01-28

**Authors:** Lokender Kumar, Nathanael Brenner, John Brice, Judith Klein-Seetharaman, Susanta K. Sarkar

**Affiliations:** ^1^Department of Physics, Colorado School of Mines, Golden, CO, United States; ^2^Quantitative Biosciences and Engineering, Colorado School of Mines, Golden, CO, United States; ^3^Department of Chemistry, Colorado School of Mines, Golden, CO, United States

**Keywords:** *Pseudomonas aeruginosa*, cephalosporins, quorum sensing (QS), biofilm, molecular docking, *Caenorhabditis elegans*

## Abstract

*Pseudomonas aeruginosa* utilizes the quorum sensing (QS) system to strategically coordinate virulence and biofilm formation. Targeting QS pathways may be a potential anti-infective approach to treat *P. aeruginosa* infections. In the present study, we define cephalosporins’ anti-QS activity using *Chromobacterium violaceum* CV026 for screening and QS-regulated mutants of *P. aeruginosa* for validation. We quantified the effects of three cephalosporins, cefepime, ceftazidime, and ceftriaxone, on (1) pyocyanin production using spectrophotometric assay, (2) bacterial motility using agar plate assay, and (3) biofilm formation using scanning electron microscopy. We also studied isogenic QS mutant strains of PAO1 (Δ*lasR*,Δ*rhlR*,Δ*pqsA*, and Δ*pqsR)* to compare and distinguish QS-mediated effects on the motility phenotypes and bacterial growth with and without sub-MIC concentrations of antibiotics. Results showed that cephalosporins have anti-QS activity and reduce bacterial motility, pyocyanin production, and biofilm formation for CV026 and PAO1. Also, sub-MICs of cefepime increased aminoglycosides’ antimicrobial activity against *P. aeruginosa* PAO1, suggesting the advantage of combined anti-QS and antibacterial treatment. To correlate experimentally observed anti-QS effects with the interactions between cephalosporins and QS receptors, we performed molecular docking with ligand binding sites of quorum sensing receptors using Autodock Vina. Molecular docking predicted cephalosporins’ binding affinities to the ligand-binding pocket of QS receptors (CviR, LasR, and PqsR). To validate our results using an infection model, we quantified the survival rate of C*aenorhabditis elegans* following *P. aeruginosa* PAO1 challenge at concentrations less than the minimum inhibitory concentration (MIC) of antibiotics. *C. elegans* infected with PAO1 without antibiotics showed 0% survivability after 72 h. In contrast, PAO1-infected *C. elegans* showed 65 ± 5%, 58 ± 4%, and 49 ± 8% survivability after treatment with cefepime, ceftazidime, and ceftriaxone, respectively. We determined the survival rates of *C. elegans* infected by QS mutant strains Δ*lasR* (32 ± 11%), Δ*rhlR* (27 ± 8%), Δ*pqsA* (27 ± 10%), and Δ*pqsR* (37 ± 6%), which suggest essential role of QS system in virulence. In summary, cephalosporins at sub-MIC concentrations show anti-QS activity and enhance the antibacterial efficacy of aminoglycosides, a different class of antibiotics. Thus, cephalosporins at sub-MIC concentrations in combination with other antibiotics are potential candidates for developing therapies to combat infections caused by *P. aeruginosa.*

## Introduction

Bacteria use quorum sensing (QS) to communicate by producing specific chemical signaling molecules and coordinating their population-wide behavior (Rutherford and Bassler, 2012). For example, *P. aeruginosa* utilizes QS for virulence factor production, cell survival, and biofilm formation (Castillo-Juarez et al., 2015). *Pseudomonas aeruginosa* is a Gram-negative pathogen responsible for frequent hospital-acquired infections of the bloodstream, the respiratory tract, and the urinary tract. QS involves the expression of acyl-homoserine lactone (AHL) molecules and detecting these signal molecules using specific protein receptors (Schütz and Empting, 2018). In the *P. aeruginosa* QS system, homo-serine lactone molecules (HSL) produced by LuxI-type enzymes bind to the cognate transcriptional regulator (LuxR-type proteins), enabling receptor dimerization and promoter binding (Li and Nair, 2012). This signal–receptor complex acts as a transcriptional regulator and initiates the expression of an array of virulence and biofilm formation associated genes (Kumar et al., 2015).

There has been significant progress in understanding the QS network of *P. aeruginosa*, which comprises three co-dependent systems, namely, las, rhl, and pqs (McKnight et al., 2000). Las and rhl systems respond to N-(3-oxododecanoyl) homoserine lactone (3O-C12-HSL) and N-butyryl homoserine lactone (C4-HSL) (Song et al., 2011). The LasR/3OC12–HSL complex also activates transcription of the rhlR system, and Rhl/C4–HSL enhances the expression of numerous genes, including the las regulon genes (Ding et al., 2018). Pqs activation results in the synthesis of 2-heptyl-3-hydroxy-4-quinolone (PQS) that connects the las and rhl QS systems. This global regulatory organization of QS allows *P. aeruginosa* to synchronize population-wide behaviors and survive under hostile environmental conditions. Emerging research has suggested that QS mutant strains make thinner biofilms and show decreased expression of virulence factors (Kumar et al., 2009).

Quorum sensing inhibition has been considered as a prospective antimicrobial approach to attenuate the virulence of *P. aeruginosa*. It can be accomplished by either signal molecule degradation or targeting the signal molecules’ interaction with their cognate receptors. Since this strategy does not directly target vital pathways required for bacterial survival, the probability of the emergence of resistant bacterial strains against anti-QS drugs is low. Thus, targeting the QS cell-signaling pathways can offer an alternative antimicrobial strategy against *P. aeruginosa* with a lower chance of developing drug resistance. There are reports on the anti-QS activity of antibacterial agents (Nazzaro et al., 2013), phytochemicals (Ding et al., 2017), and antifungal drugs (D’Angelo et al., 2018) against *P. aeruginosa*. Furthermore, halofuranes from macroalgae, specifically furanone C30, are natural anti-QS molecules that inhibit QS at relatively low doses (1–10 μM) (Hentzer et al., 2003). Most of these compounds have shown anti-QS properties at lower concentrations and antimicrobial properties at higher concentrations.

Combining antibiotics’ anti-QS and antibacterial activities is a relatively new opportunity to reduce the MIC and drug resistance probability of antibiotics. Several classes of antibiotics are useful as antibacterial agents against *P. aeruginosa* and have anti-QS activities. For example, azithromycin has anti-QS and antibacterial activities and is effective against *P. aeruginosa* infection in chronic pulmonary disorders and cystic fibrosis (Imperi et al., 2014). Tobramycin (Babić et al., 2010), ciprofloxacin (Gupta et al., 2015), and doxycycline (Husain and Ahmad, 2013) also have anti-QS activities against *P. aeruginosa.* The broad-spectrum cephalosporin antibiotics provide protective efficacy and improve the outcome of *P. aeruginosa* infections (El Solh and Alhajhusain, 2009). Cefepime (CP) (Su et al., 2017), ceftazidime (CF) (Eron et al., 1983), and ceftriaxone (CT) (Bassetti et al., 2018) have significant activities against *P. aeruginosa*. However, the emergence of drug resistance has restricted these antibiotics’ use (Akhabue et al., 2011). Consequently, the use of anti-QS antibiotics at sub-inhibitory concentrations in combination with other antibiotics may improve the outcome of infection treatment and reduce the emergence of antibiotic resistance phenotypes.

In this paper, we quantify the anti-QS effects of cephalosporins and define the mechanism of their QS inhibition using a combination of experiments and computations. First, we used the *Chromobacterium violaceum* CV026 strain to screen cephalosporin antibiotics for anti-QS activity. Prior reports have used CV026 to screen anti-QS compounds, and the selected compounds are potential anti-QS drugs against *P. aeruginosa. Chromobacterium violaceum* utilizes AHL-dependent QS pathways to produce violacein pigment, leading to purple bacterial colonies. As the negative control, we have used the CV026 mutant with an AHL synthase deletion, which appears colorless since it cannot synthesize the QS signaling molecule. However, the mutant can respond to AHL supplements and lead to purple color colonies. We found that sub-inhibitory CP, CF, and CT concentrations showed potent inhibitory effects on pyocyanin production, motility, and biofilm formation against CV026. Next, we quantified the effectiveness of these three cephalosporins against *P. aeruginosa* PAO1 and several mutants of *P. aeruginosa* with deletions of QS pathways. We confirmed that cephalosporins have anti-QS against *P. aeruginosa* and found that cephalosporins’ anti-QS activity can enhance aminoglycosides’ antimicrobial activity. The molecular docking analysis showed the possible interactions of cephalosporins with QS receptors, and provided a mechanism of anti-QS activity. To validate these findings in an infection model, we tested CP, CF, and CT antibiotics by treating *C. elegans* infected with *P. aeruginosa*. The sub-inhibitory concentrations of cephalosporins improved the survival of *C. elegans* against *P. aeruginosa* infections. As such, a combined treatment with anti-QS and antibacterial agents may provide an alternative approach in treating *P. aeruginosa* infections.

## Results

### Anti-QS Activity of Cephalosporins Against *C. violaceum* CV026

**FIGURE 1:**
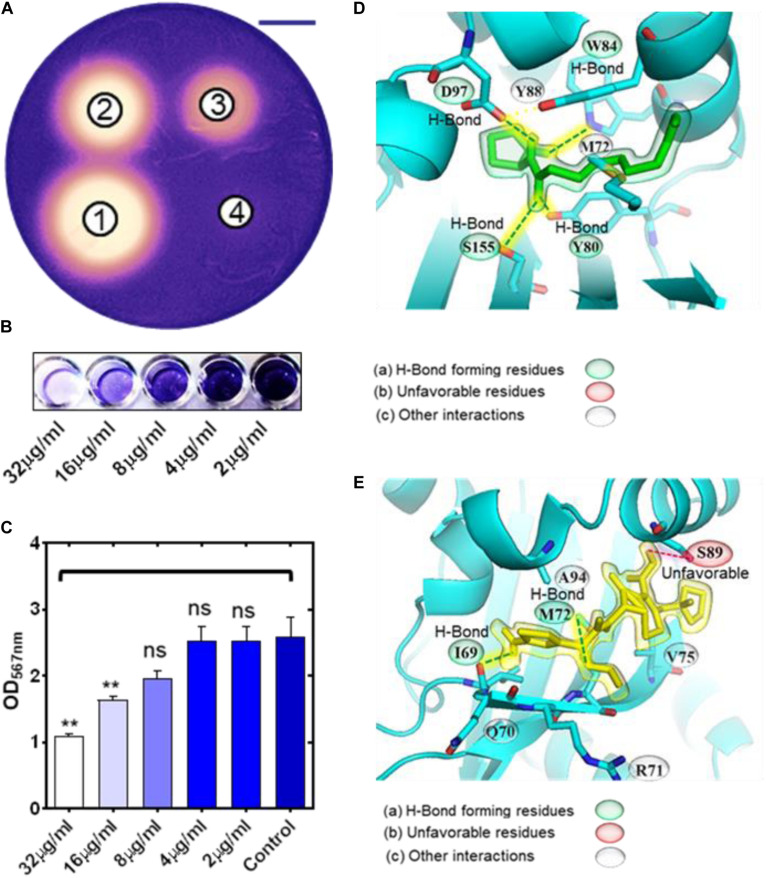
Inhibition of QS in *Chromobacterium violaceum* CV026 by cephalosporin antibiotics. Photograph of *C. violaceum* plates showing the zone of growth inhibition and pigment production inhibition (on the edge of the zones) by CP **(A)** at different concentrations (well-1 = 51.2 μg; well-2 = 25.6 μg; well-3 = 12.8 μg; well-4 = 6.4 μg) (scale bar = 20 mm). Image of microtiter plate wells **(B)** and OD_567 nm_ measurements showing the inhibition of pigment production in 96 well plate assay by sub-inhibitory concentrations of CP **(C)**. Molecular docking results showing the binding interaction of natural ligand C-10 homoserine lactone molecule **(D)**, and CP **(E)**, with CviR receptor of *C. violaceum* (ns *p* > 0.05, **p ≤ 0.01).

Results of agar well diffusion assay showed that cephalosporins displayed growth inhibitory zones reporting their antimicrobial activity against *C. violaceum* CV026 (Figure 1A and Supplementary Figure 1). In addition to the antimicrobial zone, there was a distinct halo zone of pigment inhibition at the interface of bacterial growth (purple color) and antimicrobial zone (Figure 1A). The halo zone of pigment inhibition was designated as the anti-QS zone. Each antibiotic was tested in eight dilutions (4 wells in each plate) with numbers 1-8 indicating highest to lowest concentrations of antibiotics (from 512 μg to 0.4 μg). Most of the antibiotics showed a significant zone of anti-QS activity (Supplementary Figure 2). The largest anti-QS zone was exhibited by CP (0.66 + 0.87 mm) (Figure 1A), CF (0.48 + 0.52 mm) (Supplementary Figure 3A), and CT (0.24 + 0.36 mm) (Supplementary Figure 3B). Other cephalosporin antibiotics showed a small yet significant zone (*p* < 0.05) of anti-QS activity including imipenem (0.19 + 0.07 mm), doripenem (0.26 + 0.87 mm), meropenem (0.25 + 0.13 mm) and ertapenem (0.31 + 0.09 mm) (Supplementary Figures 1D-H, 2).

Further, we determined the MICs of CP, CF, and CT to validate the anti-QS activity at sublethal concentrations against the CV026 strain. MICs of CP, CF, and CT were 128, 8, and 16 μg/mL (Supplementary Figure 5). The CV026 strain showed lower absorbance at 8, 4, and 2 μg/mL for CT as compared to the control (Supplementary Figure 5B). However, the cells were present as indicated by the visible turbidity indicating the cells survived the antibiotic treatment. CF also showed lower absorbance at 4, 2, and 1 μg/mL concentrations (Supplementary Figure 5C). Similarly, CP showed lower absorbance at 64, 32, 16, and 8 μg/mL. However there was no significant effect on the absorbance at 4, 2, 1, and 0.5 μg/mL concentrations (Supplementary Figure 5D). To quantify the anti-QS activity using pigment inhibition, we selected the 1/2th, 1/4th, 1/8th, and 1/16th concentrations of MIC values of CP, CT, and CF antibiotics. CV026 showed purple pigment production in microtiter wells with visible cell growth. Quantification of the pigment was performed by measuring the OD at 567 nm. The intensity of each well was compared with the control. CP at 32.0 μg/mL and 16.0 μg/mL significantly (*p* < 0.05) inhibited pigment production (Figures 1B,C), while CF inhibited pigment production at 2.0 μg/mL (Supplementary Figures 3C,D). CT showed significant pigment inhibition at 4.0 μg/mL (Supplementary Figures 3E,F). In these experiments, we have used sublethal concentrations of antibiotics throughout. In all cases, bacterial growth was devoid of pigment production, indicating that cephalosporins interfered with QS pathways in *C. violaceum* CVO26 with minimum effect on bacterial growth.

### Molecular Docking of Cephalosporins to the CviR Binding Pocket

We performed molecular docking of the CviR receptor with CP, CF, and CT using Autodock Vina (Figures 1D,E, Supplementary Figures 3G,H, and Table 1). To find interacting amino acids in the binding pockets of the CviR receptor, we selected amino acids within 5Å distance of each ligand. The natural ligand of the CviR receptor, C10-HSL, showed the highest predicted binding affinity with −6.9 kcal mol^–1^. The stabilization of C10-HSL in the binding pocket of the receptor is based on hydrogen bond interactions of the carbonyl group with Trp84, the amide carbonyl group with both Tyr80 and Ser155, and the secondary amine with Asp97 (Figure 1D and Table 1). The last carbon on the alkane tail of C10-HSL formed an alkyl interaction with Met72 and a π-alkyl interaction with Tyr88. CT displayed the most favorable interactions with the CviR receptor, with a predicted affinity of −6.5 kcal mol^–1^. CT formed four hydrogen bonds; one between Met72 and the carbonyl on the beta-lactam ring, one between Ile69 and the carboxylic acid, and two more hydrogen bonds with Tyr88 and Asn92 from the amine connected to the aromatic ring (Supplementary Figure 3G, Table 1). A π-sulfur interaction was formed between Met72 and the aromatic ring, an alkyl interaction between Ala94 and the non-aromatic ring thioether, as well as a π-alkyl interaction with the aromatic ring Ala94. In addition to this, an unfavorable interaction was formed between Ala94 and the proton of the amine connected to the aromatic ring, which may be the reason for the slightly lower predicted energy than the natural ligand. CP showed the second highest predicted binding affinity of -6.3 kcal mol^–1^ with the CviR receptor. CP formed π-alkyl interactions with Met72 and Ala94 and an amide-π stacking interaction with Gln70 (Figure 1E and Table 1). Two hydrogen bonds were formed between (1) Ile69 and the proton of the primary amine connected to the aromatic ring and (2) Met72 and the nitrogen in the methoxy-amino group. Alkyl interactions were formed with Met72 and Val75 residues. In addition, an unfavorable donor-donor interaction was detected between the proton of the carboxylic acid and Ser89. Among the three inhibitors, CF showed the lowest affinity for the CviR receptor (−6.1 kcal mol^–1^). CF formed a hydrogen bond with Ser89 from the primary amine connected to the aromatic ring, and another with Met72 from a carboxylic acid group. Near the carboxylic acid group, four alkyl interactions were formed with the neighboring carbon atoms (Supplementary Figure 3H, Table 1).

**TABLE 1:**
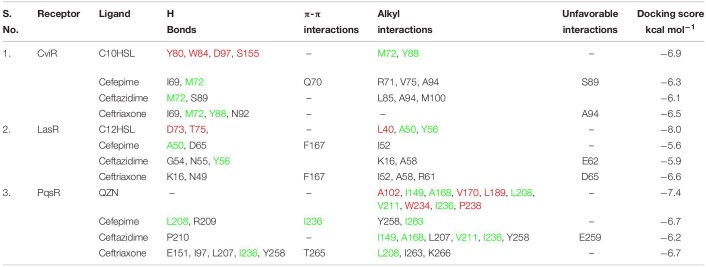
Comparative analysis of molecular interaction of natural ligands and antibiotics with receptor proteins.

*Amino acids found only in the natural binding pocket are highlighted in red, while amino acids found in the natural and the antibiotic binding pockets are highlighted in green. Amino acids only found in inhibitor binding pockets are shown in black.*

### Effect of Cephalosporins on Pyocyanin Production

**FIGURE 2:**
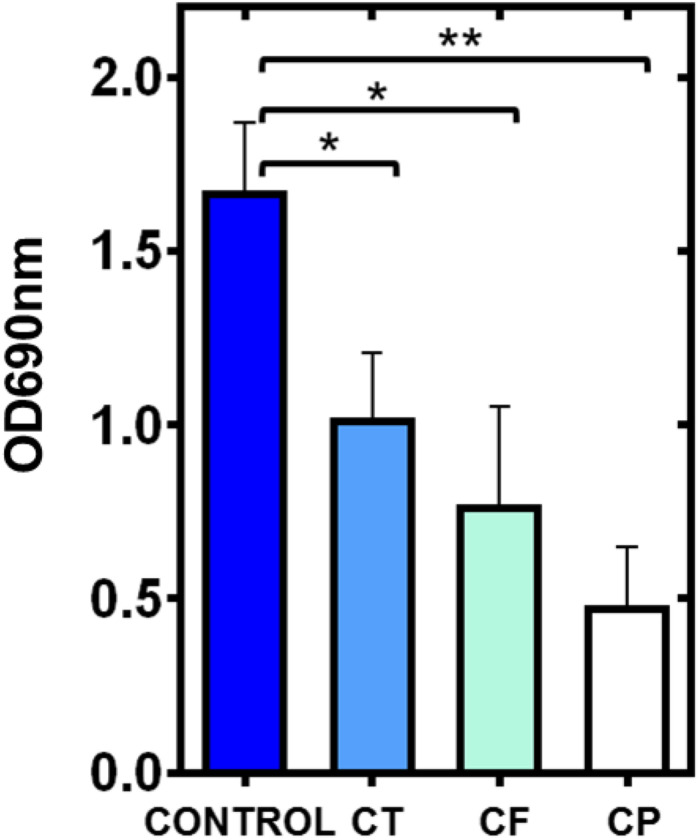
Inhibition of pyocyanin production of *Pseudomonas aeruginosa* PAO1 by cephalosporins. Graphical representation of OD_690 nm_ showing the inhibition of blue color (pyocyanin production) by *P. aeruginosa* PAO1 in the presence of CT (CT), CF (CF), and CP (CP) (*p ≤ 0.05 and **p ≤ 0.01).

The antibiotic treatment showed significant inhibition of pyocyanin production as compared to control (Figure 2 and Supplementary Figure 4). PAO1 showed significant pyocyanin pigment production OD_690 nm_ (1.67 + 0.19) and with CT (1.02 + 0.18), CF (0.77 + 0.27), CP (0.48 + 0.17). There is a clear reduction of pyocyanin production by CP, CF, and CT supplementation. These results collectively suggested that CP, CF, and CT interfered with molecular pathways associated with pyocyanin production.

### Effect of Cephalosporins on Motility Phenotypes of *P. aeruginosa* PAO1

**FIGURE 3:**
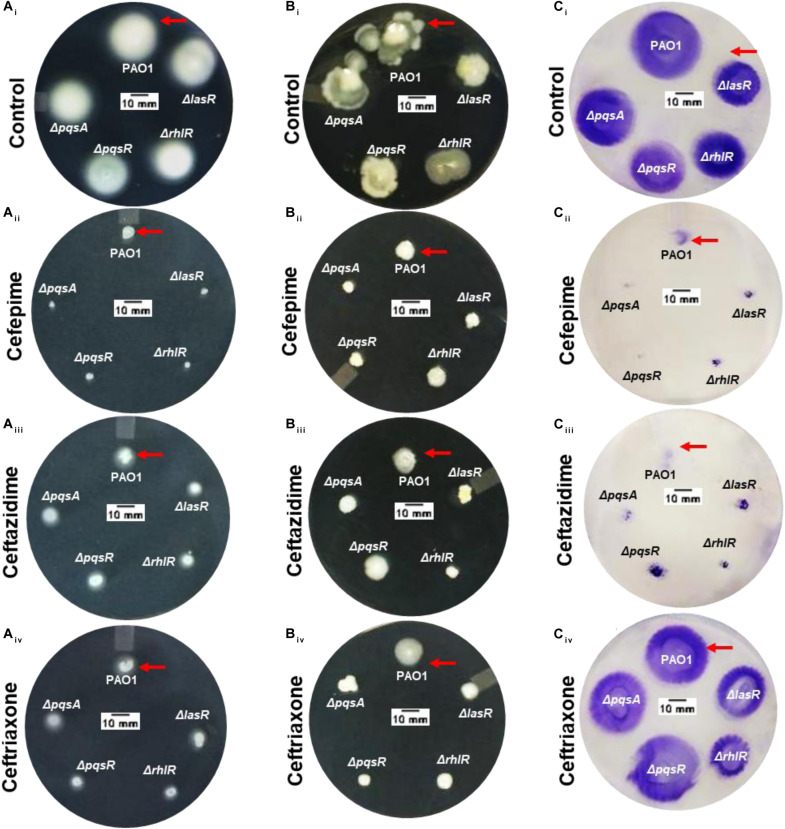
Effect of cephalosporins on motility phenotypes of *Pseudomonas aeruginosa* PAO1 and its isogenic QS mutant strains. Image showing the comparative zone of swimming **(A_i_)**, swarming **(B_i_)**, and twitching motility **(C_i_)** of PAO1 and its isogenic QS mutant strains (Δ*lasR*,Δ*rhlR*,Δ*pqsA*, and Δ*pqsR*). Image of the media plate showing the effect of CP **(A_ii_,B_ii_,C_ii_)**, CF **(A_iii_,B_iii_,C_iii_)**, and CT **(A_iv_,B_iv_,C_iv_)**, on swimming, swarming, and twitching motility zones of *P. aeruginosa* PAO1 (Scale bar = 10 mm).

**FIGURE 4:**
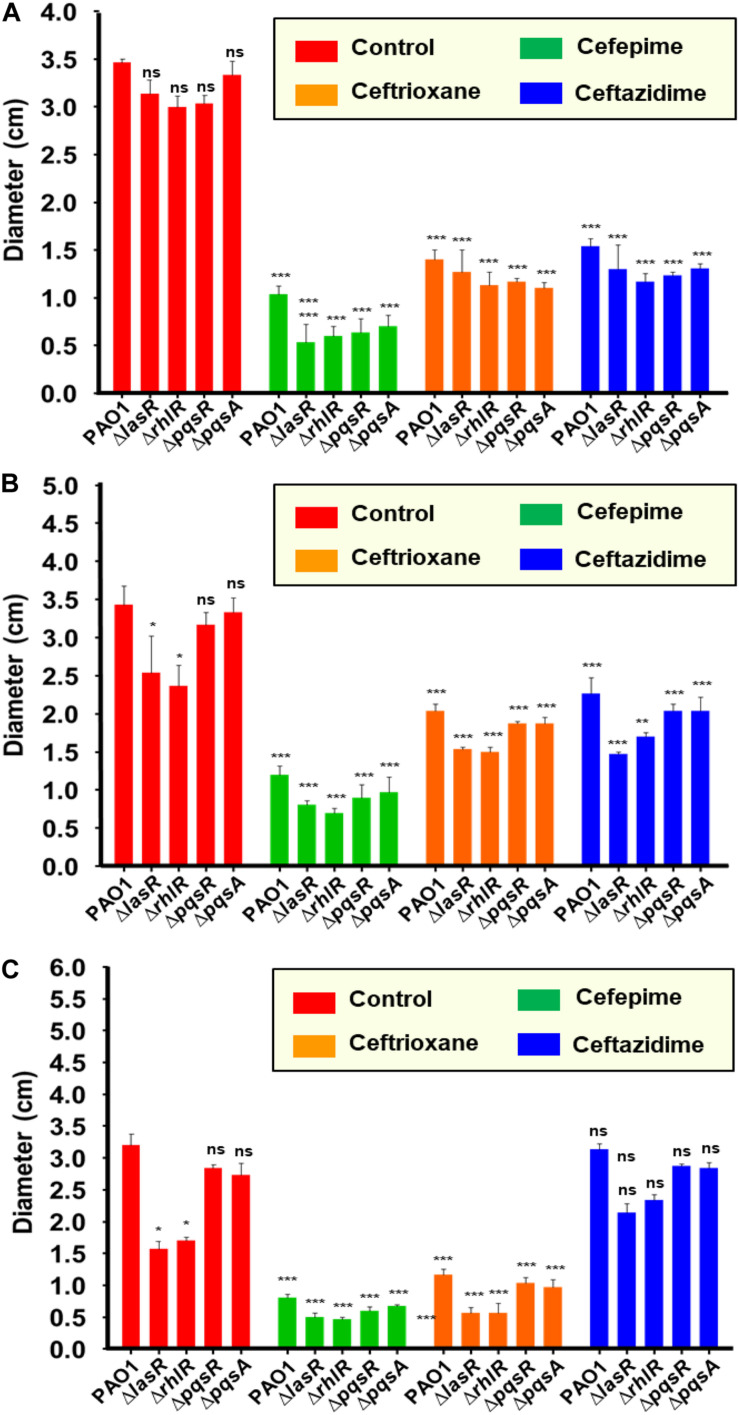
Quantification of motility inhibition (diameter of motility zones) of *Pseudomonas aeruginosa* by cephalosporins: Comparative analysis of the diameter of the motility zone (mm) for PAO1, Δ*lasR*,Δ*rhlR*,Δ*pqsR* and Δ*pqsA* in presence of CP **(A)**, CF **(B)** and CT **(C)**. Not significant (ns): *p* > 0.05, *p ≤ 0.05, **p ≤ 0.01, ***p ≤ 0.001.

Motility phenotypes were analyzed for PAO1 control and QS mutant strains. We found that the QS mutant strains showed no significant difference in swimming motility, which suggests that the flagella-dependent swimming motility is independent of QS pathways (Figures 3A_**i**_, 4A). Interestingly all three antibiotics CP, CF, and CT showed significant swimming motility inhibition (*p* < 0.001) of PAO1 as well as the isogenic QS mutant strains (Figure 4A). The diameters of the zones are mentioned as table insert in Figure 4A. Further, results showed that the Δ*lasR* and Δ*rhlR* mutant strains showed a significant difference (*p* < 0.05) in swarming motility as compared to the control indicating that the motility is dependent on las and rhl QS pathways (Figures 3B_**i**_, 4B). The pqsR and pqsA mutant strains showed no significant difference in the swarming motility zone as compared to the control PAO1. Interestingly all three antibiotics CP, CF, and CT, showed significant swarming motility inhibition (*p* < 0.001) for wild-type PAO1 and mutant strains (Figures 3B_**i**_, 4B). Effect on twitching motility was also analyzed on PAO1 and its isogenic mutant strains, and it was found that the Δ*lasR/*Δ*rhlR* mutant strains showed significant difference (*p* < 0.05) in the twitching motility (Figures 3C_**i**_, 4C), indicating that the twitching motility is dependent on las and rhl QS pathways. Interestingly only two antibiotics CP and CT, showed significant twitching motility inhibition (*p* < 0.001), but surprisingly, CF showed no motility inhibition (Figures 3C_**i**_, 4C). Our results showed that CP and CF have a strong inhibitory effect on motility phenotypes.

### Anti-biofilm Activity of Cephalosporins Against *P. aeruginosa* PAO1

**FIGURE 5:**
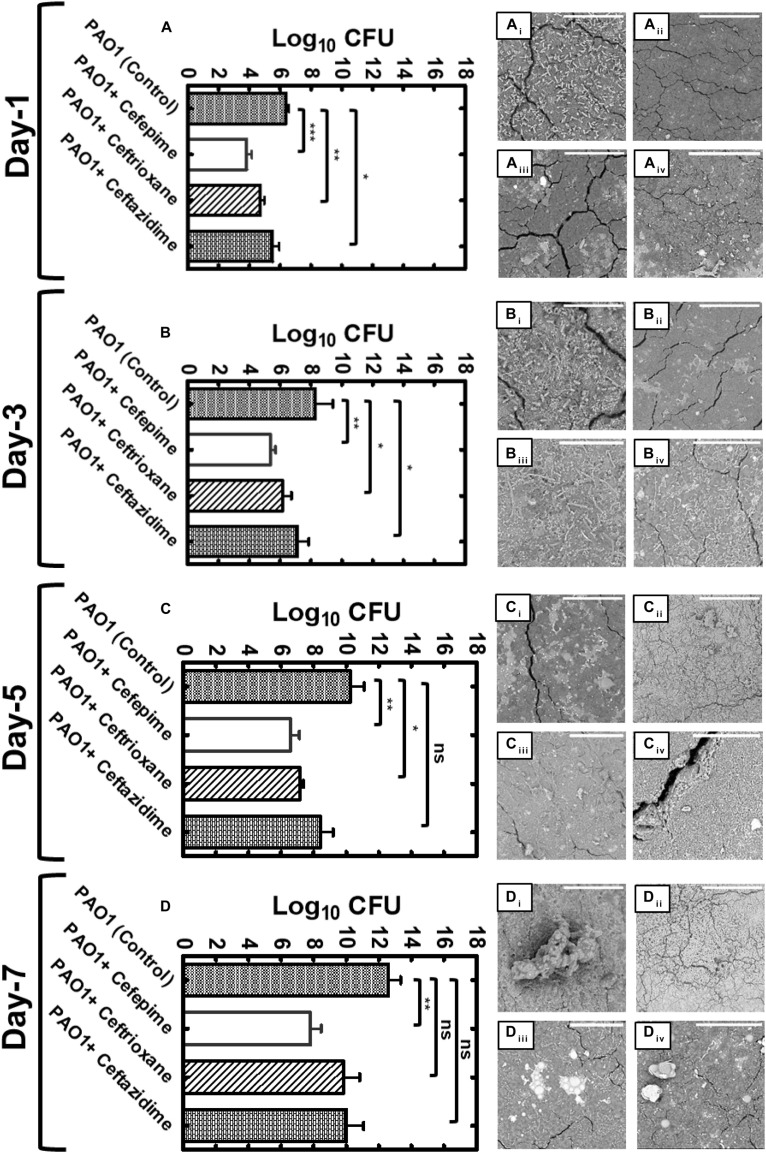
Effects of QS inhibition by cephalosporins on biofilm formation by PAO1. Graphical representation of live bacterial cell quantification of *Pseudomonas aeruginosa* PAO1 on urinary catheter surface under sub-MIC concentrations of CP, CT and CF for day-1 **(A)**, day-3 **(B)**, day-5 **(C)** and day-7 **(D)** (ns *p* > 0.05, *p ≤ 0.05, **p ≤ 0.01, ***p ≤ 0.001). Scanning Electron Microscope (SEM) images of biofilms formed by *P. aeruginosa* PAO1 on the urinary catheter in the presence of CP, CF, CT on day-1 **(A_**ii**_,B_**ii**_,C_**ii**_,D_**ii**_)**, day-2 **(A_**ii**_,B_**ii**_,C_**ii**_,D_**ii**_)**, day-3 **(A_**iii**_,B_**iii**_,C_**iii**_,D_iii_)** and day-4 **(A_**iv**_,B_**iv**_,C_**iv**_,D_**iv**_)** (Scale bars represent 50 μm).

We used Environmental Scanning Electron Microscopic (ESEM) to image biofilms produced by PAO1 (Figure 5). ESEM imaging and live cell counts were performed on every alternate day (days 1, 3, 5, and 7) on the urinary catheter surface. On day 1, PAO1 control biofilms showed the presence of a uniform monolayer of cells attached to the urinary catheter surface. Cells were uniformly distributed within the extracellular polysaccharide matrix (Figure 5A_**i**_). The live cell count of the day 1 biofilm was high (6.39 ± 1.89 log cfu) (Figure 5A). In antibiotic-treated groups, CP showed low cell counts with significantly less polysaccharide matrix distributed throughout the catheter surface (Figure 5A_**ii**_). CP treatment significantly reduced (p < 0.001) the log cfu count as compared to the control (3.83 ± 0.32 log cfu). CF and CT both showed a significant amount of exopolysaccharide distributed on the catheter surface (Figures 5A_**iii**_,A_**iv**_). Both antibiotics showed reduced live cell counts of 4.70 ± 0.26 and 5.50 ± 0.43 log cfu (Figure 5A). Three-day-old-biofilms of PAO1 of the control group showed the formation of a thick layer of bacterial cells embedded in an exopolysaccharide matrix (Figure 5B_**i**_). Biofilms appeared to be complex in structure with the presence of long polymer threads, mucilaginous matrix, and high cellular growth. Live cell counts were higher on day 3 in control groups (8.28 ± 1.13 log cfu) (Figure 5B). The CP-treated catheter surface showed low cell counts attached to the urinary catheter surface at day 3 (Figure 5B_ii_). The bacterial live cell count on day 3 in CP treated urinary catheters was lower (5.01 + 0.56 log cfu) as compared to the control group (Figure 5B). CF and CT thin biofilm formation on the catheter surface (Figures 5B_**iii**_,B_**iv**_). The bacterial cells appeared to be longer in size as compared to the control. Log cfu count of the treated groups were significantly reduced (*p* < 0.05) as compared to the PAO1 control (CT 6.0 ± 0.56 log cfu and CF 6.6 ± 0.73 log cfu) (Figure 5B). Five-day-old-biofilms of the PAO1 control group showed thick biofilm (Figure 5C_**i**_). On day 5, the biofilms started to construct mushroom-shaped structures. At this stage, biofilms might be completely resistant to antimicrobial agents due to the protection provided by the exopolysaccharide layer. These clusters of live bacterial cells help in the formation of the three-dimensional architecture of bacterial biofilm. As expected, the biofilm showed a high live cell count in five-day-old biofilms (10.05 ± 0.86 log cfu) (Figure 5C). In CP treated groups, the biofilm appeared to be defective (Figure 5C_**ii**_), and the log cfu count of the live cell was significantly lower, indicating the potent antibiofilm activity of CP (Figure 5C). In CT and CF, treated biofilms were condensed and covered with thick polysaccharide matrix on the catheter surface (Figures 5C_**iii**_,C_**iv**_). However, there was no significant difference in the log cell count of live cells in CF treated biofilms as compared to the control group. CT treated biofilms showed a significant difference in the log cfu count of bacterial cells (7.01 ± 0.19 log cfu) (Figure 5C). Moreover, on day-7 control biofilms appeared highly complex three dimensional with numerous mushroom-shaped secondary structure on the biofilm surface (Figure 5D_**i**_). In the untreated control group, biofilms showed a larger assembly of multi-mushroom shaped structures, and the log cfu count of live cells in 7-day-old-biofilm was found to be very high (12.60 ± 0.75 log cfu). The seven-day-old CP treated biofilms were still defective in biofilm architecture. The mushroom-shaped structures were absent, and individual cells can be visualized on the catheter surface. In treated biofilms, bacterial cells appeared as long thread shaped and produced significantly less polysaccharide with a low log cfu count of 7.81 ± 0.65 (Figure 5D). CT and CF biofilms were covered with mushroom-shaped structures, and few individual cells were visible, indicating the thick three-dimensional biofilm formation. Live cell count of the catheter surface of these antibiotic-treated groups was found to be non-significant as compare to control (Figure 5D).

### Combinatorial Effect of Cefepime and Aminoglycosides Against *P. aeruginosa* PAO1

**FIGURE 6:**
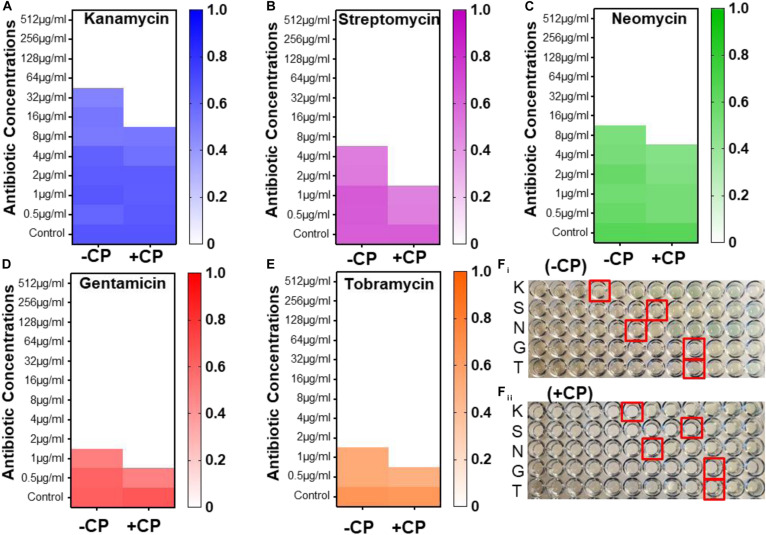
Synergistic antimicrobial effects of CP and aminoglycosides against *Pseudomonas aeruginosa* PAO1. Heat map of OD_600 nm_ of *P. aeruginosa* PAO1 with kanamycin **(A)**, streptomycin **(B)**, Neomycin **(C)**, Gentamicin **(D)**, and Tobramycin **(E)** in presence and absence of CP. Image of microtiter plate showing the MIC of aminoglycosides against *P. aeruginosa* PAO1 in the 96-microtiter plate with and without CP **(F_**i**_,F_**ii**_)**. (Red rectangular boxes in the image represents the MIC of aminoglycosides against PAO1-wells with no visible bacterial growth); K, kanamycin; S, streptomycin; N, Neomycin; G, Gentamicin; and T, Tobramycin.

**FIGURE 7:**
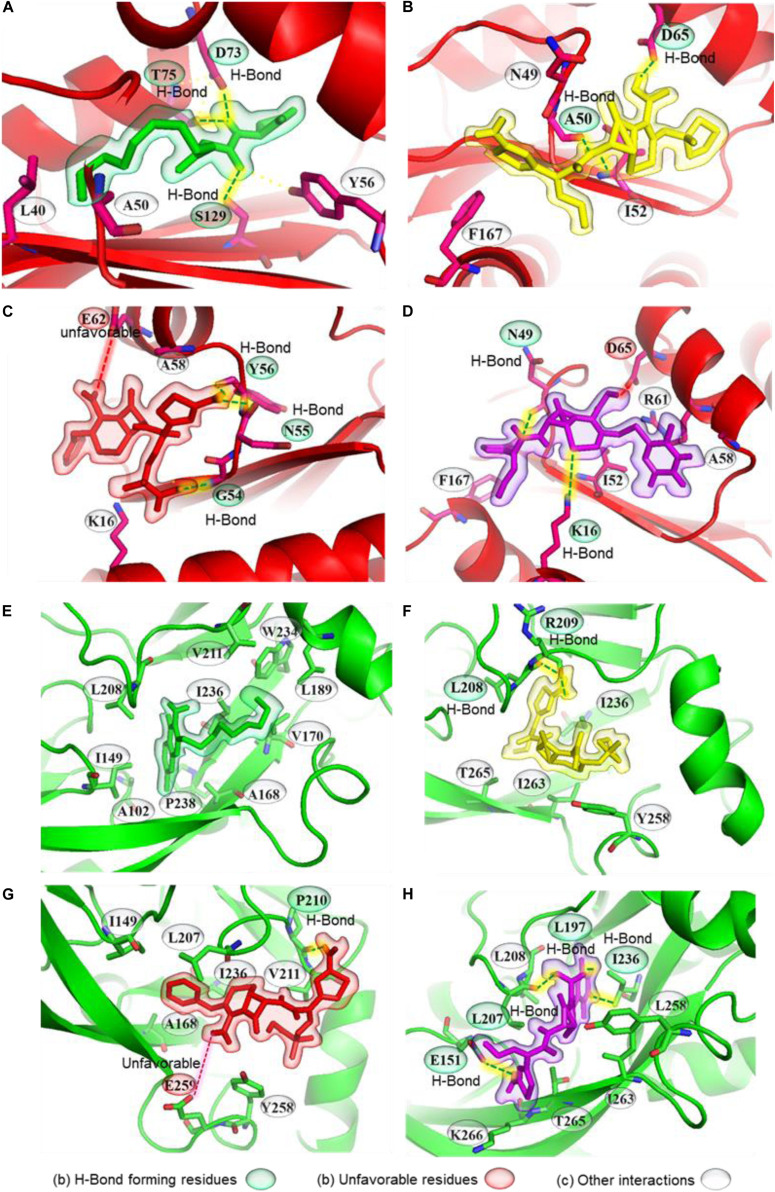
Molecular interactions of natural ligand and cephalosporin antibiotics with *Pseudomonas aeruginosa* QS receptors (LasR and PqsR). Representation of ligand-receptor interactions of 3oxo-C12-HSL **(A)**, CP **(B)**, CF **(C)**, CT **(D)** with LasR QS receptor. Representation of ligand-receptor interactions of Pseudomonas quinolone signaling (PQS) molecule **(E)**, CP **(F)**, CF **(G)**, CT **(H)** with PqsR QS receptor. LasR and PqsR receptors are represented as red and green. Natural ligands of receptors, CP, CF, and CT, are presented as green, yellow, red, and purple.

First, the antimicrobial efficacy of aminoglycosides was tested against *P. aeruginosa* PAO1. Kanamycin sulfate showed poor antibacterial activity against *P. aeruginosa* PAO1 (MIC: 64 μg/mL); however, in the presence of CP, the MIC of kanamycin has significantly decreased to 16 μg/mL (Figure 6A). Streptomycin sulfate showed potent efficacy against PAO1, and the MIC was found to be 8 μg/mL (Figure 6B). In combination with CP, the MIC of streptomycin was significantly reduced to 2 μg/mL. Similarly, the MIC of neomycin was found to be 16 μg/mL, and in combination with CP, the MIC was decreased to 8 μg/mL (Figure 7C). Gentamicin and tobramycin were found to be highly effective antibiotics against PAO1 with the MIC values of 2 μg/mL (Figures 6D,E). However, in combination with CP, the MIC values were decreased to 1 μg/mL. Our results showed that the CP at a sub-inhibitory concentration in combination with major aminoglycosides led to the increased antimicrobial potency of aminoglycosides. This combination needs to be tested in animal models to validate its effectiveness against *P. aeruginosa* and could be established as a clinically useful combinational therapy to treat *P. aeruginosa* infections.

### Molecular Docking of Cephalosporins With *P. aeruginosa* Quorum-Sensing Receptors

We docked 3-oxo-C12HSL to its binding pocket in the LasR receptor. The predicted binding affinity was −8.0 kcal mol^–1^. 3-oxo-C12HSL formed three hydrogen bonds, one with Ser129 and the amide carbonyl, and two more with Asp73, Thr75, and the proton of the secondary amine (Figure 7A and Table 1). A hydrophobic interaction connected Tyr56 and the ring carbon neighboring the secondary amine, supported by two alkyl interactions between the carbon at the end of the hydrophobic tail with Leu40 and Ala50. Next, the three selected antibiotics were docked to the same ligand-binding pocket of LasR. CP formed two hydrogen bonds, one between the carboxyl group and Asp65 and another between Ala50 and the proton of the secondary amine connected to the beta-lactam ring (Figure 7B). A π- sulfur interaction was formed between Phe167 and the sulfur atom of the aromatic ring, an electrostatic interaction was formed between Asp65 and the charged nitrogen atom, a carbon-hydrogen bond was formed between Asn49 and the beta-lactam carbonyl, and an alkyl interaction was formed between Ile52 and the thioether group. CP showed a comparative docking score of −5.6 kcal mol^–1^. CF formed three hydrogen bonds; one between Gly54 and a carboxyl group, and two between the primary amine with Tyr56 and Asn55. Lys16 formed a carbon-hydrogen bond with the carboxyl group and an alkyl interaction with a nearby methyl group (Figure 7C and Table 1). A π - alkyl interaction was formed between Ala58 and the aromatic ring, and an unfavorable electrostatic interaction was formed between Glu62 and the carboxyl nearest to the beta-lactam ring. CF showed a comparatively low docking score of −5.9 kcal mol^–1^. CT formed two hydrogen bonds; one between Asn49 and the primary amine and another between Lys16 and the non-aromatic ring thioether (Figure 7D and Table 1). A π - sulfur interaction was formed between Phe167 and the aromatic ring thioether. Carbon hydrogen bonds were formed between Ala58 and the methyl group connected to the triazine ring; and between Asn49 and the beta-lactam carbonyl. Three alkyl interactions were formed; one from Ile52 to the non-aromatic thioether ring, two from the triazine ring to Ala58 and Arg61, respectively. CT showed a comparatively higher docking score of −6.6 kcal mol^–1^.

Molecular docking was also performed independently with the PqsR receptor. The natural ligand formed no hydrogen bonds but formed ten alkyl interactions between its two rings with Leu208, Ala168, Ile149, Pro238, and Ala102, as well as five alkyl interactions between the last carbon in the hydrophobic tail and Leu189, Val170, Trp234, Val211, Ile236 of PqsR receptor (Figure 7E and Table 1). The natural ligand showed a docking score of −7.4 kcal mol^–1^. CP formed two hydrogen bonds from its primary amine to Arg209 and Leu208. A carbon-hydrogen bond was formed between the methoxyl group and Thr265 (Figure 7F and Table 1). Two π-σ interactions were formed from the aromatic ring to Leu208 and Ile236. An alkyl interaction and a π-alkyl interaction were formed from the thioether to Ile263 and Tyr258, respectively. The docking score of CP was found to be −6.7 kcal mol^–1^. Similarly, CF formed a hydrogen bond between its primary amine and Pro210 as well as a carbon-hydrogen bond from Leu207 to the six-membered ring (Figure 7G and Table 1). An unfavorable interaction was also formed between Glu259 and the carboxyl nearest the beta-lactam ring. CF showed a docking score of −6.2 kcal mol^–1^. CT formed four hydrogen bonds; one between its primary amine and Glu151, one between Leu207 and the carboxyl group, one between Ile236 and the proton on the triazine ring, and lastly, one between Leu197 and a carboxyl on the triazine ring (Figure 7H and Table 1). The docking score of CT was −6.7 kcal mol^–1^. These conformations would obstruct the entry into the binding pocket, and therefore may interrupt ligand-receptor interactions.

### Protective Effect of Cephalosporins in the *C. elegans* Infection Model

**FIGURE 8:**
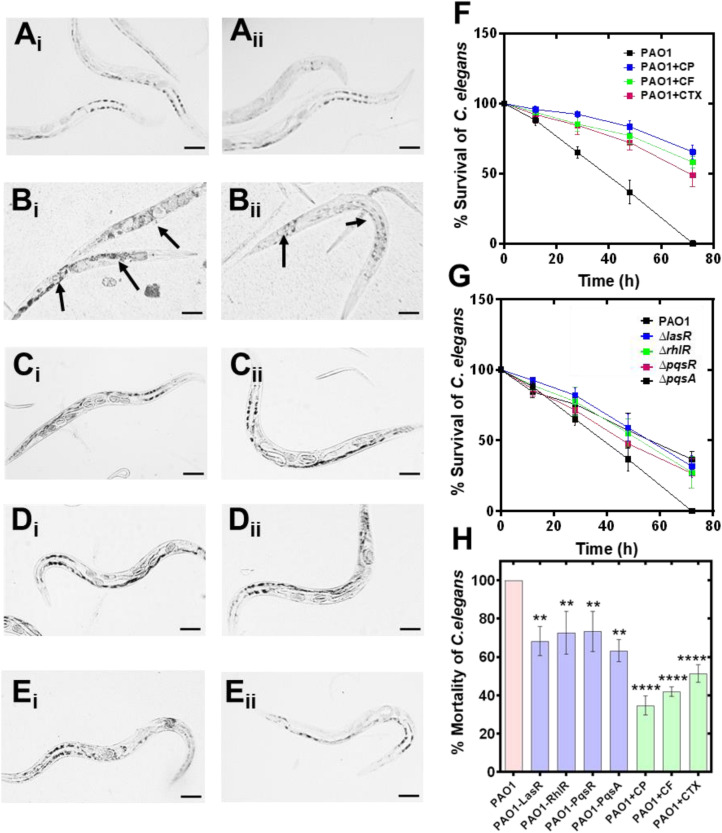
Anti-virulence effect of cephalosporins against *Pseudomonas aeruginosa* PAO1 induced tissue damage in *Caenorhabditis elegans*. Light microscopic images of *C. elegans* showing the protective effect of cephalosporin in *P. aeruginosa* mediated tissue damage; control *C. elegans* (healthy) **(A_i_,A_ii_)**; *C. elegans* exposed to PAO1 **(B_i_,B_ii_)**; PAO1 + CP **(C_i_,C_ii_)**; PAO1 + CF **(D_i_,D_ii_)**, and PAO1 + CT **(E_i_,E_ii_)**. Percentage survival of PAO1 infected *C. elegans* in the presence of CP, CF, and CT **(F)**. Percentage survival of *C. elegans* by the QS mutant strains (Δ*lasR*,Δ*rhlR*,Δ*pqsR*, and Δ*pqsA*) and control PAO1 strain **(G)**. Comparative analysis of the % mortality of *C. elegans* after 72 h by control PAO1 strain, isogenic QS mutant strains (Δ*lasR*,Δ*rhlR*,Δ*pqsR*, and Δ*pqsA*), and after treatment with CP, CF, and CT **(H)**. **p ≤ 0.01, ****p ≤ 0.0001.

We have used *C. elegans* as an animal model to investigate the protective effect of CP, CF, and CT against *P. aeruginosa* PAO1. Advantage of its transparent nature, internal organs can be visualized directly. In healthy worms, the intestine, uterus, proximal and distal gonads (Figures 8A_**i**_,A_**ii**_) were found to be intact, and no tissue damage was observed. The diseased/dead worm has blunt-body curvature with no/reduced body movement; these were distinct phenotypic characteristics of the tissue damage or diseased state of the *C. elegans* (Dimov and Maduro, 2019). PAO1 infected *C. elegans* showed potent damage of intestinal tissue after 72 h leading to 100% mortality in infected worms. Percentage survival of *C. elegans* at 12, 24, and 28 h was 88.0 + 3.60%, 65.0 + 4.30% and 36.6 + 8.30% and at 72 h, it has achieved 100% mortality (Figure 8F). Interestingly, the sub-inhibitory concentration of antibiotic treatment showed a protective effect in *C. elegans* infected groups. CP treatment showed a significant increase in the percentage survival of *C. elegans.* At 12, 24, 48, and 72h the percentage survival was 95.6 + 2.51%, 92.33 + 2.50%, 83.33 + 4.16%, and 65.33 + 5.03% (Figure 8F). Similarly, CF treated groups also showed a significant increase in worm survival. At 12, 24, 48, and 72 h the % survival was found to be 93.6 + 1.52%, 85.33 + 5.03%, 77.0 + 5.56% and 58.0 + 4.30% (Figure 8F). CT showed a similar protective effect, and treated groups showed reduced mortality. At 12, 24, 48, and 72 h, the percentage survival was found to be 92.0 + 2.60%, 84.33 + 5.56%, and 48.66 + 8.08% (Figure 8F). All three antibiotics showed a significant reduction in the *C. elegans* mortality, indicating the protective effect of a sub-inhibitory concentration of cephalosporin antibiotics. We also tested the effect of quorum sensing mutant strains on *C. elegans* survival. Percentage survival of **Delta*lasR* mutant at 12, 24, 48 and 72 h was 92.66 + 1.52%, 82.0 + 5.29%, 59.0 + 9.53% and 31.66 + 11.23% (Figure 8G). Similarly, Δ*rhlR* mutant strain showed 89.0 + 3.60%, 77.6 + 10.20%, 55.0 + 10.0% and 27.33 + 7.63% (Figure 8G). Similarly, Δ*pqsR* and Δ*pqsA* mutant strains were also showed 86.33 + 5.13%, 71.33 + 6.11%, 47.66 + 11.23%, 26.66 + 10.40% and 84.33 + 4.04%, 75.66 + 7.76%, 57.0 + 12.53%, and 36.66 + 5.77% survival rates of *C. elegans* (Figure 8G). We have also used an uninfected group with *E.coli* OP50 to check the *C. elegans* mortality, and no mortality was observed until 72 h. The result of our experiment showed that the sub-MIC concentration of antibiotics protected *C. elegans* from *P. aeruginosa* infection and led to an increase in the survival of the worm. Finally, we have compared the percentage mortality of all the groups with the control PAO1 group, and all the groups have shown a significant reduction in the percentage mortality at 72 h (Figure 8H). Additionally, mortality rates were higher in the QS mutant strains as compared to the antibiotic-treated groups (Figure 8G). This indicates that cephalosporins might have additional alternative mechanisms apart from their anti-QS activity to protect *C. elegans* from *P. aeruginosa* infection.

In the future, it might be interesting to investigate the effect of these antibiotics at the molecular level against *P. aeruginosa.* This may lead to the identification of novel molecular pathways in *P. aeruginosa.*

## Discussion

Antibiotics show hormesis effect, and in addition to their antimicrobial activity, they can influence alternate pathways at the molecular level in bacterial cells. Research showed that the long-term anti-QS macrolide azithromycin therapy in *P. aeruginosa* infected patients has led to the dramatic improvement in clinical symptoms and respiratory. Currently, there is a huge intellectual gap in our understanding of alternative mechanisms of antibiotics. In the present study, we focused on evaluating the anti-quorum sensing activity of cephalosporins against *P. aeruginosa* at sub-inhibitory concentrations. We have utilized the CV026 strain in agar well diffusion assay to screen the anti-QS activity of major β-lactam antibiotics. Our results showed that β-lactams showed potent antimicrobial activity against *C. violaceum* (Figure 1, Supplementary Figures 1, 2, 3). In a typical agar well diffusion assay, the concentration of antibiotics decreases due to diffusion from wells towards the periphery. At the interface between bacterial growth and no growth, there is a zone where antibiotic is present in sub-inhibitory concentrations. The Sub-MIC is not sufficient to kill the bacteria; however, it can provide useful information on the alternative activity of antibiotics such as anti-QS activity. *C. violaceum* CV026 is an AHL negative strain that does not synthesize AHL signal molecules, but this strain responds to exogenous AHL molecules (from C4 to C8 acyl side chains AHL molecules) (Devescovi et al., 2017). This assay provides information on (1) antimicrobial activity and (2) anti-QS activity of tested compounds.

Three major cephalosporins showed a large zone of pigment inhibition at the interface of growth and no growth indicating their potential anti-QS activity against *C. violaceum* CV026. The presence of the zone of pigment inhibition indicated that the cephalosporin antibiotics were interfering with the activation of the QS pathway in the CV026 strain (Figure 1, Supplementary Figures 1, 2, 3). It was clear that carbapenem (MP, IP, DP, and EP) showed significantly low anti-QS activity as compare to cephalosporins (CP, CF, and CT). We tested sub-MICs to find out the concentration that induces a significant anti-QS effect in CV026. The high anti-QS activity of CP might be because of the high MIC of CP, and the antibiotic is available in high sub-MIC. Based on our results, we excluded carbapenems and selected cephalosporins (CP, CF, and CT) due to their high anti-QS activity.

Computational biology provides an excellent tool to understand ligand-receptor interactions at the atomic level (King et al., 2016). Molecular docking, in particular, can characterize the molecular interactions between small molecules and the target proteins. We used this technique to gain insights into cephalosporin-CviR receptor interactions. The active site of CviR is occupied by its natural ligand (C10HSL), and it is buried deep inside the ligand-binding domain of the CviR receptor (Figure 1D). The active site is not fully accessible to cephalosporins due to their relatively large size. However, molecular docking revealed that the cephalosporins might have the affinity to bind with amino acids at the edge of the natural binding pocket, including Met72 and Tyr88 (Figure 1E, Supplementary Figures 2G,H). This binding might block ligand binding or may induce conformational changes to block activation of CviR. Molecular docking results showed CF and CT also showed unfavorable interactions with Ser89 and Ala94. The docking score predicted the affinity of cephalosporins against CviR (higher to lower) as CT > CP > CF. Our experimental anti-QS trend followed the sequence CP > CT > CF. In both cases, CF showed the lowest activity, suggesting the conclusion that CF binding is the weakest of the three ligands. However, in molecular docking experiments, CT showed the highest score. This may indicate that there might be other factors, including conformational dynamics and drug bioavailability, that may contribute to the anti-QS activity. Further, the effect of antibiotics supplementation on *P. aeruginosa* virulence factors production was evaluated.

Pyocyanin is a blue-colored secondary metabolite and major virulence factor responsible for the cell toxicity of *P. aeruginosa* (Lau et al., 2004). Previous research has shown that the pyocyanin deficient phnAB and PhzB1 mutants produce a low level of pyocyanin, leading to reduced mortality in the burn wound model of *P. aeruginosa* (Lau et al., 2004). Pyocyanin also induces pro-inflammatory and pro-oxidant responses leading to host cell lysis (Arora et al., 2018). Therapy targeting pyocyanin production in *P. aeruginosa* may suppress tissue damage during infection. Our results showed that all three antibiotics significantly suppressed the pyocyanin production *in vitro*, and their ability in the suppression of pyocyanin production was as follows: CP > CF > CT (high to low) (Figure 3 and Supplementary Figure 4). Previously anti-QS effect of macrolide antibiotics (azithromycin and erythromycin) has been related to pyocyanin inhibitory effect (Köhler et al., 2010).

Cellular motility is responsible for the spread of infection and assists bacterial cells to translocate toward nutrient-rich environments (Arora et al., 2005). *P. aeruginosa* is a motile Gram-negative bacterium, and motility phenotypes significantly influence the establishment and spread of infections. We already mentioned that the QS mutant strains showed no significant difference in the swimming motility (Figures 3, 4). Interestingly, CP, CF, and CT antibiotics showed motility inhibition of wild type PAO1 and its isogenic QS mutant strain. This provides a clue that cephalosporins might have a direct effect on flagellum proteins. Compound targeting swimming motility may inhibit surface colonization leading to defective biofilm formation. Inhibition of swimming motility by cephalosporins may target surface colonization and biofilm dispersal by *P. aeruginosa.* Previous research has shown that the anti-motility compounds target gene synthesis of flagellum and pilus synthesis (Navidifar et al., 2019) to inhibit the movement of *P. aeruginosa* (Rashid and Kornberg, 2000). Swarming motility is also a flagella-dependent movement of cells to spread as a biofilm over biotic and abiotic surfaces (Pallaval Veera et al., 2018). Previous research has shown that QS controls biofilm development by regulating swarming motility phenotypes (Shrout et al., 2006). Our results also showed that swarming motility is dependent on las and rhl signaling systems and may be independent of the PQS signaling system. The inhibition of swarming motility also provides strong evidence that cephalosporins may interfere with the QS communication of *P. aeruginosa.* Twitching motility is a flagella-independent bacterial motion on moist surfaces by extension and retraction of polar type IV pili of *P. aerugionsa* (Mattick, 2002). Previously, researchers debated the dependence of twitching motility on QS pathways (Beatson et al., 2002). However, it is known that twitching motility is required for the establishment of infection and biofilm formation. Our results showed that las and rhl systems were involved in pilli-mediated twitching motility. There was also a significant difference in the twitching motility of **Delta*lasR* and **Delta*rhlR* mutant strains with and without cephalosporins. In contrast, CT showed no effect on twitching motility phenotypes of PAO1 or its isogenic mutant (Figures 3, 4). This indicates that the cephalosporins except CT have a direct effect on pilli function. The future research require indepth understanding of the molecular mechanism of antibiotics on flagellar and pilli proteins and quantifying the effect on cell movements.

Bacterial biofilms are complex three-dimensional bacterial colonies that are resistant to antibiotic treatment (Stewart and Costerton, 2001). Previous research has shown that the las QS system is required for the maturation of *P. aeruginosa* biofilms (Davies et al., 1998), and it plays an important role in biofilm development stages. The rhl QS also plays a key role in biofilm formation, and it can control biofilm formation independent of its canonical HSL autoinducer activation pathway (Mukherjee et al., 2017). The PqsR mutation has been linked with reduced biofilm formation in *P. aeruginosa* (Wu and Yan, 2019). The regulatory role of las, rhl, and pqs system in biofilm formation provides an exciting opportunity to develop anti-QS therapy as potential antibiofilm agents. Our results indicated that the cephalosporins show an anti-biofilm effect at sub-inhibitory concentrations. SEM analysis and quantitative live bacterial count showed the following pattern of antibiofilm efficacy of cephalosporins: CP > CF > CT (Figure 5). This pattern was similar to the anti-QS activity pattern. In addition, despite the low anti-QS activity, the inability of CT to inhibit twitching motility might also contribute to its low antibiofilm efficacy. CP was the most effective antibiofilm agent and significantly inhibited *P. aeruginosa* biofilms. However, CF was ineffective against day-five, and CT was ineffective against day-five and day-seven old biofilms. In the future, it would be interesting to monitor the biofilm eradication effect of cephalosporins on established biofilms. In addition, cephalosporin treatment has significantly reduced the exopolysaccharide matrix production in early to late biofilm stages. Qualitative SEM analysis showed that the CP was found to be the most effective treatment to reduced exopolysaccharide matrix production even in late biofilm stages. The exopolysaccharide matrix helps the cells during initial attachment and regulates the transition from reversible to irreversible attachment biofilm development. In the future, quantitative estimation of alginate, extracellular DNA, and other matrix proteins might precisely quantify the comparative effect of cephalosporins on exopolysaccharide matrix production. Our results suggested the pattern of inhibition as follows: CP > CF > CT (high to low) (Figure 5). CP showed the highest antibiofilm activity against *P. aeruginosa* biofilms.

A combination of antibiotic treatments against bacterial pathogens is preferred due to its high potency. Our results showed that CP has significantly decreased the MIC of aminoglycosides (Figure 6). There was 75% decrease in kanamycin MIC (from 32 μg/mL to 8 μg/mL) and streptomycin MIC (from 4 μg/mL to 1 μg/mL) in presence of CP. Neomycin, gentamicin, and tobramycin showed a 50% decrease in MIC values in the presence of cefepime. Our results suggested that although CP enhanced the antimicrobial activity of all the tested aminoglycosides; however, it worked better with kanamycin and streptomycin (Figure 6). Further research is required to validate its efficacy in animal models to confirm the *in vitro* results. Various studies have shown the combinatorial effect of antimicrobial agents against *P. aeruginosa.* However, their mechanism of combinatorial activity is poorly understood. Previous studies have shown that the combination of CF with tobramycin increases the antimicrobial potency against *P. aeruginosa* (Dundar and Otkun, 2010). Gentamicin and ciprofloxacin, in combination, have also been reported effective against *P. aeruginosa* (Wang et al., 2019).

The docking score of cephalosporins to LasR showed the following pattern: CT > CP > CF. This docking pattern was similar to the docking score of CviR, and CT showed the highest binding affinity to the LasR receptor. The LasR docking scores are low as compared to the CviR receptor docking scores indicating a low binding affinity for LasR. The LasR natural ligand-binding pocket is buried deep inside the receptor and hence most likely inaccessible to the cephalosporins due to their large size (Figure 7). However, the antibiotics showed significant interactions with amino acids surrounding the binding pocket. This suggests that the cephalosporins might be able to block the entry of the ligands to the binding pockets, thereby inhibiting the activation of LasR. CT and CF showed single unfavorable interactions with the LasR receptor. CP, CT formed two hydrogen bonds, and CF formed three H-bonds without any unfavorable interactions with the LasR receptor. Interestingly, CT showed the highest docking affinity with the lowest anti-virulence effect *in vitro.*

Our docking results with PqsR showed the following pattern: CP = CT > CF. All three cephalosporin showed high docking scores with the receptor. The docking scores of CP and CT were found to be equal. However, CF showed one unfavorable interaction, and that might have contributed to its slightly lower docking score as compared to CP and CT. Moreover, the docking score of QZN was −7.4, and the ligand was not forming any hydrogen bonds with the receptor. CP formed a hydrogen bond with leu208 and π-interactions with Ile236 (Figure 7). It is important to note that these amino acids were also contributing towards alkyl interactions with QZN indicating CP was able to bind to the active site of LasR. Similarly, CT formed hydrogen bonds with Ile236, and CF formed alkyl interactions with Ile236, which is also shared by QZN. This clearly indicates that all three antibiotics were interacting with a common target area on PqsR. The high docking score of CP with PqsR was consistent with its potent pyocyanin inhibition activity. Previous reports have shown that the pqs system controls pyocyanin production. The high-predicted binding affinities for cephalosporins with LasR and PqsR receptors may indicate that their binding plays an important role in interfering with the QS system of *P. aeruginosa.*

*Caenorhabditis elegans* has been used as an established animal model to investigate the virulence of *P. aeruginosa* (Tan et al., 1999), and *C. elegans* has been widely used as an animal model system to investigate anti-infective activities of synthetic and natural compounds against various bacterial pathogens (Patel et al., 2019; Rajkumari et al., 2019). Our results suggest that the wild type PAO1 strain achieved 100% mortality after 72 (Figure 8). On the other hand, we have found that QS mutant strains showed significantly high *C. elegans* surviving score (%) as follows: Δ*pqsA* (36.7 ± 5.8%) > Δ*lasR* (31.7 ± 11.2%) > Δ*rhlR* (27.3 ± 7.6% %) > Δ*pqsR* (26.7 ± 10.4%). Zaborin et al. showed that phosphate depletion in *P. aeruginosa* activates phosphate signaling (PhoB), expresses pyoverdin, and activates MvfR-PQS systems causing the death of *C. elegans* and mice. Tan et al. demonstrated that several *P. aeruginosa* factors (toxA, 34H4, 25F1, dsbA, gacA, toxA, and plcS) are required for the killing of the mammalian as well as the plant host. *P. aeruginosa* mediated worm mortality is induced by cyanide asphyxiation and paralysis (Gallagher and Manoil, 2001). The cyanide production in *P. aeruginosa* is controlled by the *hcn* operon and regulated by LasR and RhlR QS systems (Pessi and Haas, 2000).

Our results showed that all mutant strains were defective in virulence, and *C. elegans* survived the infection by QS mutant strains; however, the protection was not comprehensive since the survivor rate was low with all the mutant strains. Δ*pqsA* and Δ*lasR* mutants showed the highest surviving, although the score with Δ*rhlR* and Δ*lasR* mutant was also significantly high. Treatment of *C. elegans* with sub-MICs of cephalosporins has shown a significant protective effect on the worm’s mortality. For CP, CF, and CT, the worm survival score at 72 h was 65.3 ± 5.0%, 58.0 ± 4.3%, and 48.7 ± 8.1% (CP > CF > CT) (Figure 8). Previous research has shown that QS inhibitors protect *C. elegans* and human lung epithelial cells from *P. aeruginosa* (O’Loughlin et al., 2013). Papaioannou et al. showed that quorum sensing quencher enzyme PvdQ increases the life span of the *C. elegans* and reduces the pathogenicity of *P. aeruginosa*. PvdQ degrades quorum sensing signal molecules (AHLs), leading to the disruption of quorum sensing and the suppression of QS-regulated virulence factors. Anti-QS effects of clove oil have been shown to reduce las- and rhl-regulated virulence factors such as *LasB*, protease, and pyocyanin production, motility, and exopolysaccharide production, and protect *C. elegans* against *P. aeruginosa* infection (Husain et al., 2013). The anti-QS activity of 2, 5-Piperazinedione has been linked in the proteolytic and elastolytic activities of PAO1 and significant protection of *P. aeruginosa* pre-infected *C. elegans* (Musthafa et al., 2012a). The anti-QS activity of antagonistic compound phenylacetic acid (PAA) has been shown to suppress QS-dependent exopolysaccharide, pyocyanin, elastase, and protease production in PAO1, and treatment of PAO1-infected *C. elegans* showed enhanced survival after treatment with PAA (Musthafa et al., 2012b). The protective effect of CP may be linked with its potent anti-QS, anti-biofilm and anti-pyocyanin activity against *P. aeruginosa.* It is important to consider that in our study, none of the antibiotics (at sub-inhibitory concentrations) showed complete protective effect at the sub-MIC concentration (at 72 h), indicating that the anti-QS therapeutics may protect from acute infection progression, however for complete treatment of infection combinatorial therapies are needed. Further research is needed to identify the pathways or strategies to ‘switch off’ the virulence mechanism leading to complete protection from the infection.

## Conclusion

We demonstrated that the cefepime, ceftazidime, and ceftriaxone show anti-QS, biofilm inhibition, and anti-pyocyanin activity against *P. aeruginosa*. Hence, these antibiotics may provide a novel structure scaffold for the development of effective QS inhibitors. We also showed that cefepime improves the ability of *C. elegans* to survive *P. aeruginosa* infection. Therefore, cefepime may prove to be a valuable antibiotic to develop novel treatment strategies against biofilm-associated *P. aeruginosa* infections.

## Materials and Methods

### Bacterial Strains

*Chromobacterium violaceum* CV026, *P. aeruginosa* wild type strain PAO1 and its defective QS mutant strains including Δ*lasR*,Δ*rhlR*,Δ*pqsA*, and Δ*pqs*R were kindly provided by Dr. Paul Williams (Professor of Molecular Microbiology, Faculty of Medicine and Health Science, University of Nottingham, United Kingdom). *C. violaceum* CV026 was cultured using Luria Broth with kanamycin at 50 μg/mL (30°C) under stationary conditions and used for the detection of acyl-homoserine lactone and screening of anti-QS activity of β-lactam antibiotics. All bacterial strains were maintained as 25% glycerol stocks and stored at −80°C. Fresh subculture was performed from frozen stocks and used for each experiment.

### Antibiotics and Chemicals

The following antibiotics cefepime hydrochloride (#PHR1763-1G:LRAB8503, sigma), ceftriaxone disodium salt hemi-heptahydrate (#C5793-1G: 027M4799, sigma), ceftazidime (#CDS020667-50MG:B02487097), oxacillin sodium salt (Cat#28221-5G:097M4853V), imipenem (#PHR1796-200MG:LRAB7177, sigma), doripenem hydrate (#SML1220-50MG:025M4717V, sigma), meropenem trihydrate (#USP-1392454: 50K434), ertapenem (#CDS022172-25MG:B02467020) were procured in dry powder and stored in 4^*o*^C under desiccated conditions. Master stocks of 1024 μg/mL were made in sterile distilled water and stored in -80^*o*^C for future use. Every time fresh stock of antibiotics was used for experiments. All the other chemicals are of ACS analytical grade.

### Screening of Anti-QS Activity of Antibiotics

#### (a) Agar Well Diffusion Assay

Agar well diffusion assay using *C. violaceum* CV026 as biosensor strain was performed to screen anti-QS activity of antibiotics (*n* = 3) (Supplementary Figure 9). 50 μL of C6-HSL stock solution (10 mg/mL) was mixed with 10 mL of an overnight grown culture of *C. violaceum* CV026 (OD_600 nm_ = 1.0) and designated as AHL-CV026 solution. To prepare media plates, we slowly mixed 19 mL of tryptose soya agar (1%) with 1 mL of AHL-CV026 solution (avoid air bubble formation). Plate the mixture in a Petri plate and let the media cool down until agar is solidified. Use sterile pipette tip (200 μL) to puncher holes in solid agar. Press the pipette and twist to take the punched agar and invert the plate to remove the agar block. Discard the pipette tip and use fresh tips every time to make clean wells. Load different concentrations of antibiotics in each well and incubate the plates for 15 h at 37°C in a bacteriological incubator. We diluted the stock solution (1024 μg/mL) of antibiotic 10 fold and loaded 50 μL of each dilution in each well. The amount of antibiotics in well-1 to well-8 was as follows, well-1 = 51.2 μg; well-2 = 25.6 μg; well-3 = 12.8 μg; well-4 = 6.4 μg; well-5 = 3.2 μg; well-6 = 1.6 μg; well-7 = 0.8 μg and well-8 = 0.4 μg. After incubation, observe the plate in light back growth. Clear zone without bacterial growth and no pigment indicated antimicrobial zone and hazy zone with bacterial growth but without pigment production indicate anti-QS activity of antibiotics. The picture was taken by a Bio-Rad imager and compiled in one image with a scale bar (20 mm). Anti-QS zones were quantified following equation:

AQS Zone = (**D**_Growth+*No Pigment*_ - **D**_No Growth/No Pigment_) [AQS Zone = Anti-QS zone (cm), **D**_Growth+*No Pigment*_ = Diameter of growth zone with no pigment production, **D**_No Growth/No Pigment_ = Diameter of no growth zone and no pigment].

#### (b) Microtiter Plate Assay

Briefly, a round-bottom microtiter plate well was filled with 100 μL of LB media (*n* = 3 for each antibiotic dilution). The overnight culture of CV026 was inoculated in 10 mL of LB media and incubated for 3–4 h until the OD_600 nm_ reached 0.2–0.3. After incubation, we harvested cells and washed them three times with phosphate buffer saline (0.01 M, pH-7.4). The washed cells were reconstituted in PBS, and OD was adjusted to 0.1 (OD_600 nm_). Stock cell suspension (10 μL) was used as inoculum for each well. C6-HSL at the final concentration of 10 μg/mL was used for pigment production in each well. Antibiotics were serially diluted in the well. After adding C6-HSL and CVO20, the plate was incubated at 37°C for 24 h. After incubation, the OD_567 nm_ was measured using a plate reader. We have tested sub-MICs of CP, CF, and CT using a microtiter plate assay. The minimum inhibitory concentration was determined using National Committee for Clinical Laboratory Standards (NCCLS) (2002) guidelines (explained in section 2.4).

### MIC Determination and Sub-MIC Selection for *P. aeruginosa* PAO1 Strain

The MIC of antibiotics was determined, according to National Committee for Clinical Laboratory Standards (NCCLS) (2002) guidelines against the standard *P. aeruginosa* wild type strain, PAO1. Round bottom, 96 microwell plate was used for MIC determination. Briefly, the stock solution was serially diluted (2-fold) in cation adjusted Muller Hinton broth, and 2–8 × 10^5^ cfu was used as inoculum in the total reaction of 100 μL per well in cation Muller Hinton broth (*n* = 3 for each antibiotic dilution. After 15 h, resulting in no visible growth was selected as the MIC. Sterile media with bacteria and sterile media with antibiotics are served as positive controls and negative controls. The final plate was also read at OD_600 nm_ to quantify the effect of the antibiotic on PAO1. MIC, 1/2th, and 1/4th MIC were tested on the growth curve profile of PAO1. Respective concentrations showing no significant effect on the growth profiles were selected for anti-virulence factors and anti-biofilm assays against *P. aeruginosa*.

### Motility Competence Assay

*Pseudomonas aeruginosa* PAO1 wild type and QS mutant strains (Δ*lasR*,Δ*rhlR*,Δ*pqsA, and*Δ*pqsR*) were assessed for motility competence in the presence of a sub-inhibitory concentration of CT, CF, and CP.

#### (a) Swarming Motility Competence

Media plates were prepared with nutrient agar (8 g/L) complemented with glucose (5 g/L) in the presence of sub-MICs (1/4th MIC) of CT CF and CP. Plates were point inoculated with a sterile toothpick by an overnight culture of *P. aeruginosa* PAO1 and QS mutant strains (Δ*lasR*,Δ*rhlR*,Δ*pqsA, and*Δ*pqsR*). Swarming Motility competence was determined by measuring circular turbid zones after incubation at 37°C for 24 h.

#### (b) Swimming Motility Competence

1% tryptone, 0.5% NaCl, and 0.3% agarose media plates with sub-MIC (1/4th MIC) of CT CF and CP were prepared. Plates were point inoculated with a sterile toothpick from an overnight culture of *P. aeruginosa* PAO1 and QS mutant strains (Δ*lasR*,Δ*rhlR*,Δ*pqsA, and*Δ*pqsR*). After incubation at 37°C for 24 h, swimming motility was determined by measuring the radius of the circular pattern of bacterial migration.

#### (c) Twitching Motility Competence

Luria agar plates containing sub-MIC (1/4th MIC) of CT CF and CP were prepared. Plates were stabbed with sterile toothpick up to the bottom of the plates by an overnight culture of *P. aeruginosa* PAO1 and QS mutant strains (Δ*lasR*,Δ*rhlR*,Δ*pqsA, and*Δ*pqsR*). The plates were incubated at 37°C for 24 h, the media layer was discarded, and the hazy zone of growth at the interface between the agar and polystyrene surface was stained with 1% crystal violet stain for 10 min. Plates were washed three times with 5 mL of phosphate buffer saline (pH-7.4, 0.1 mM). After drying, the violet-colored zone of bacterial growth was measured.

### Biofilm Inhibition Estimation of Antibiotics

#### (a) Scanning Electron Microscopy

Biofilms were grown on a Foley catheter (urinary catheter) surface as follows. Urinary catheters were cut into 10 mm pieces under sterile conditions. Catheter pieces were incubated in 40 mL of Luria broth in a 50 mL falcon tube with 100 μL of PAO1 standard strain overnight culture. Catheter pieces were incubated in the presence of sub-MIC of CT CF and CP for seven days under the stationary condition at 37°C. Each group has four subsets of the experimental group for days 1, 3, 5, and 7. Each day catheter pieces were analyzed using Phenom pro-scanning electron microscope.

#### (b) Colony Forming Units (CFU) Quantification

Catheter pieces in different groups were processed as follows for quantification of live bacterial load. Each catheter piece was dipped in 1 mL phosphate buffer saline (pH-7.4, 0.1 M), and bacterial cells were scraped from the inner and outer surface of the catheter using a sterile scalpel blade. Serial dilution of the sample was performed and plated on Luria agar plates and plated were incubated at 37°C for 15 h.

### Effect of Antibiotics on Pyocyanin Production

Pyocyanin was estimated using the method of Huerta et al. [34] with slight modification. Briefly, PAO1 was grown under different concentrations (10% to 100%) of Luria broth, and different concentrations were tested for pyocyanin production (*n* = 3 for each antibiotic). At the different concentrations of antibiotics (sub-MIC) of CT CF and CP, the production of pyocyanin production was estimated and compared with control. Chloroform was added to cell-free culture supernatant at different concentrations, and OD at 690 nm of the chloroform layer was measured.

### Determination of the Combinatorial Effect of Aminoglycoside and Cephalosporins

Minimum inhibitory concentration concentrations of aminoglycosides, namely, gentamicin sulfate (Sigma, Cat# G1914), neomycin sulfate (Sigma, Cat# PHR1491), streptomycin sulfate (Sigma, Cat# S6501), tobramycin sulfate (Sigma, Cat# T1783), kanamycin sulfate (Sigma, Cat# 60615) were determined using standard 96 well plate assay as mentioned previous section in detail. To determine CP’s combinatorial effect with an aminoglycoside, we added CP at a sub-inhibitory concentration (0.5 μg/mL) in each well with different dilutions of aminoglycosides. The MIC was determined by visual detection of bacterial growth and optical density measurement of each well. OD_600 nm_ values were plotted as a heat map using Graph pad prism 8.0.

### Molecular Docking Studies

Molecular docking studies were performed using Autodock Vina (Version 1.5.6) following standard protocols. The crystal structures of CviR (PDB ID: 3QP8), LasR (PDB ID: 3IX3), and PqsR (PDB ID: 4JVI) were downloaded from the protein databank, at http://www.pdb.org. Ligands, ions, water molecules, and small molecules were removed, and the file was saved in pdbqt format using autodock tools. Ligand structure files in PDB format were downloaded from www.rcsb.org. Structures that were not available in PDB format were converted from sdf using an online converter^1^. The binding site was located by highlighting key amino acids on the receptor protein in autodock tools. Then, a grid box was placed to encase the binding site area while minimizing the total volume. A config.txt file was created to specify the receptor, ligand, and the x, y, and z dimensional coordinates of the docking site. The coordinates for protein receptors were provided as in Supplementary Table.1). For extensive simulation, the ‘exhaustiveness’ option was set as 10.0. Autodock Vina was run, and the best docking conformations were visualized using Pymol (Zalman 3D) software and BIOVIA Discovery studio. Docking conformations of each ligand were analyzed by examining their total energy score. Docking scores of cephalosporin antibiotics were compared with the natural ligand of each receptor.

### *Caenorhabditis elegans* Survival Assay

Nematode infection model (Musthafa et al., 2012b; Husain et al., 2013) with modification was used to access the anti-virulence effect of cephalosporins. *C. elegans* N2 strain was procured for CGC. The worms were cultivated in an NGM Petri plate with a bacterial lawn of OP50 cells. L2 and L3 stage nematode (20–40 worms) were transferred to 10 NGM plates to grow enough worms for the experiment. After the 4-5 days growth, the worms were collected in liquid NGM media, and the concentration of worms was adjusted to approx. 100 worms/mL (n = 100 for each group). *P. aeruginosa* PAO1 was grown at 40% LB media at 37°C stationary conditions with and without sub-inhibitory concentrations of CP, CF, and CT. The young adult nematodes were infected with PAO1 (control and antibiotic-treated) and incubated at 25°C for 15 h. After the incubation, worms were centrifuged to remove free bacteria and transferred to fresh liquid NGM media with and without the sub-inhibitory concentration of cephalosporins. QS mutant strain was also collected and processed similarly to evaluate its effect on nematode killing. Worms were confirmed dead if they did not show any moment under the microscope. Every 15 h, worms were immediately observed under a light microscope for the mortality estimation. The number of dead worms was counted, and the percentage mortality was calculated as follows: Survival (%) = Total no of worms-Number of dead worms/Total no of worms X100.

### Statistical Analysis

All experiments were performed in triplicate and repeated on different days. The effect of antibiotic treatment on pigment production, pyocyanin, motility, live cell count in biofilms, and percentage survival/mortality of *C. elegans* in different groups was evaluated using a two-way ANOVA test. The p values were calculated, and *p* < 0.05 was considered significant. Results were analyzed using Graph Prism 8.0 software. Values were expressed as mean ± standard deviation (SD).

## Data Availability Statement

The raw data supporting the conclusions of this article will be made available by the authors, without undue reservation.

## Author Contributions

LK initiated the project and designed the experiments and performed molecular dynamics simulation experiments. LK, JB, and NB performed the experiments. LK and NB performed molecular docking. LK, JK-S, and SS performed the analysis of MD simulation data. LK and SS designed the experiments on synergistic effects of antibiotics. SS developed quantitative analysis of growth curves. LK, NB, JB, JK-S, and SS analyzed the manuscript data. LK, NB, JK-S, and SS wrote the manuscript. All authors edited the manuscript.

## Conflict of Interest

The authors declare that the research was conducted in the absence of any commercial or financial relationships that could be construed as a potential conflict of interest.

## Footnotes

1 https://cactus.nci.nih.gov/translate/
