# Supplementary material for: Cephalosporins Interfere With Quorum Sensing and Improve the Ability of Caenorhabditis elegans to Survive Pseudomonas aeruginosa Infection
Source: Front Microbiol. 2021 Jan 28;12:598498. doi: 10.3389/fmicb.2021.598498 (PMC7876323; doi:10.3389/fmicb.2021.598498)
Supplement: Supplementary file 1 [file Data_sheet_1.pdf]

Supplementary information

Cephalosporins interfere with quorum sensing and improve the ability of *Caenorhabditis elegans* to survive *Pseudomonas aeruginosa* infection

Lokender Kumar[1,*], Nathanael Brenner[2], John Brice[1], Judith Klein-Seetharaman[2,3], and Susanta K. Sarkar[1,*]

[1]Department of Physics, Colorado School of Mines, Golden, CO, USA

[2]Quantitative Biosciences and Engineering, Colorado School of Mines, Golden, CO, USA

[3]Department of Chemistry, Colorado School of Mines, Golden, CO, USA

*Corresponding authors:

lokenderkumar@mines.edu and ssarkar@mines.edu

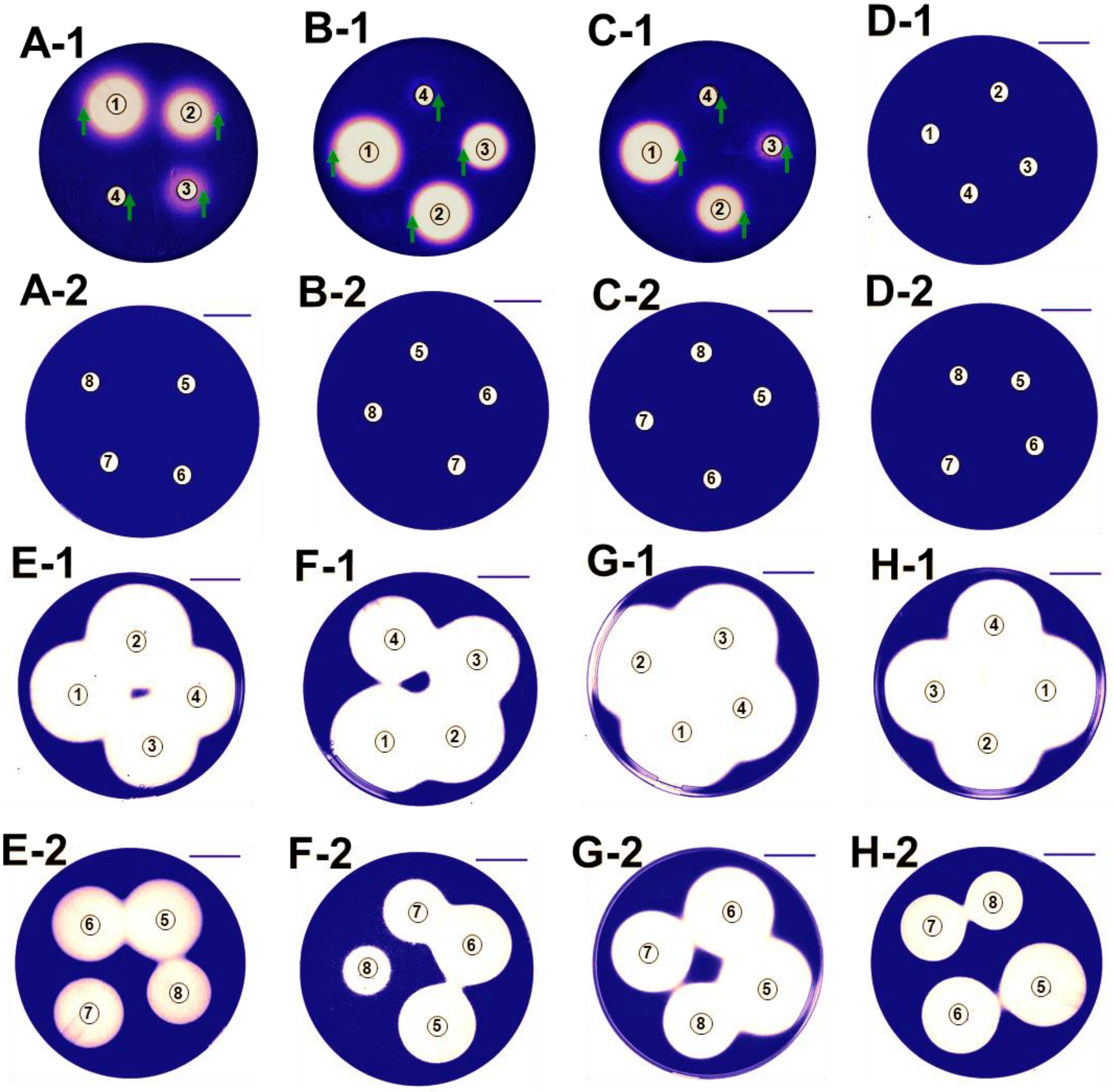

Supplementary figure-1: Screening of anti-QS activity of cephalosporin antibiotics using *Chromobacterium violaceum* CV026 as detector strain. Photographs of plates showing zone of growth inhibition and pigment production inhibition (on the edge of the zones) by different classes of antibiotics against *Chromobacterium violaceum* CV026 (Cefepime-A-1, A-2; Ceftazidime-B-1, B-2; Ceftriaxone-C-1, C-2; Oxacillin-D-1, D-2; Imipenem-E-1, E-2; Doripenem-F-1, F-2; Meropenem-G-1, G-2; Ertapenem-H-1, H-2. The green arrow indicates the presence of an anti-QS zone of inhibition (no growth inhibition) for cefepime, ceftazidime, and ceftriaxone (Scale bar 20 mm). In each plate there are eight wells; each well represents different concentration of antibiotics as follows, well-1= 51.2 µg; well-2=25.6 µg; well-3= 12.8 µg; well-4=6.4 µg; well-5=3.2 µg; well-6= 1.6 µg; well-7=0.8µg and well-8= 0.4 µg.

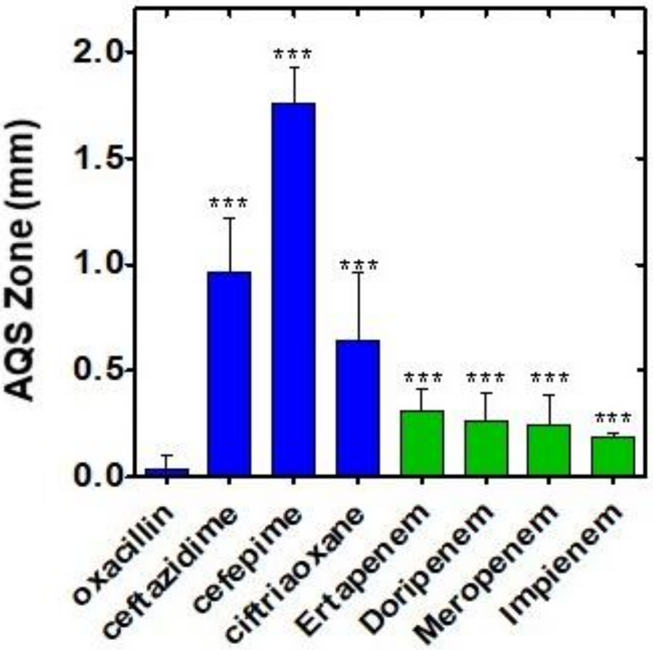

Supplementary figure 2: Comparative quantitative analysis of diameters of anti-QS activity of various antibiotics against *C. violaceum* CV026 in agar well diffusion assay. Graphical representation of the anti-QS zone in cm (the area where pigment production was inhibited but the growth of bacteria was present) in agar well diffusion assay. The diameters were compared with the anti-QS zone of oxacillin for the determination of statistical significance. Oxacillin showed no antimicrobial activity against *C. violaceum* CV026. *$p<0.05$, **$p<0.01$ and ***$p<0.001$)

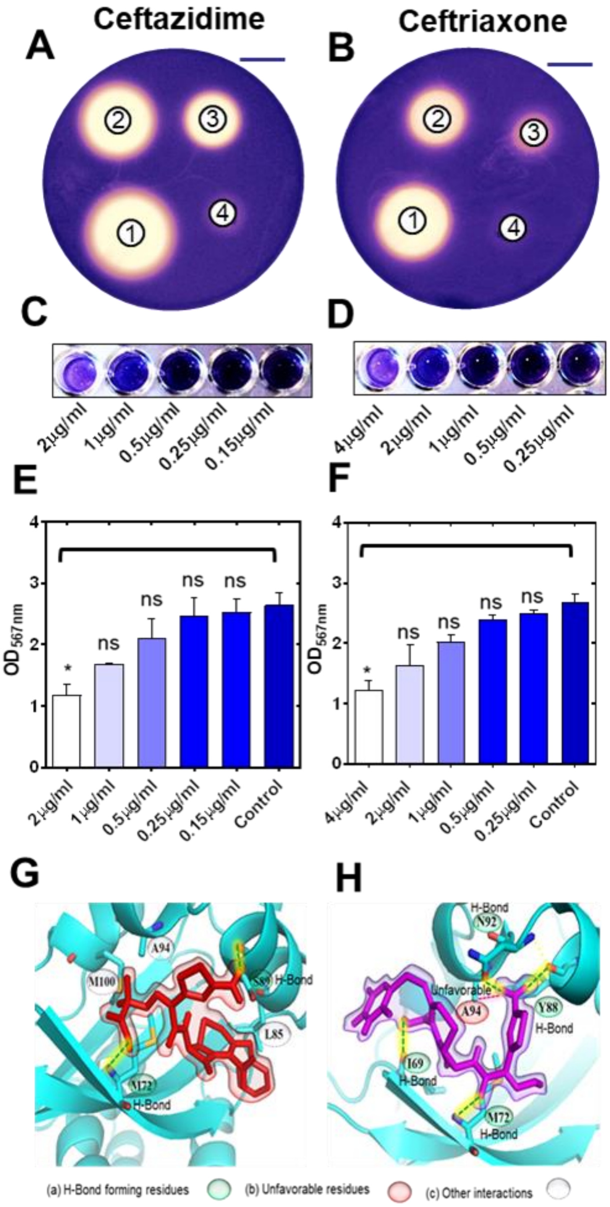

Supplementary figure 3: Inhibition of QS in *Chromobacterium violaceum* CV026 by cephalosporin antibiotics. Photographs of *C. violaceum* plates showing the zone of growth inhibition and pigment production inhibition (on the edge of the zones) by CF (A) and CT (B) against *C. violaceum* CV026 (well-1= 51.2 µg; well-2=25.6 µg; well-3= 12.8 µg; well-4=6.4 µg) (Scale bar= 20mm). Images of microtiter plate wells and OD_{567nm} measurements showing the inhibition of pigment production in 96 well plate assay by sub-inhibitory concentrations of CF (C, D) and CT (E, F). Molecular docking results showing the binding interaction of CF (G) and CT (H) with the CviR QS receptor of *C. violaceum* (ns $p > 0.05$, * $p \leq 0.05$, ** $p \leq 0.01$).

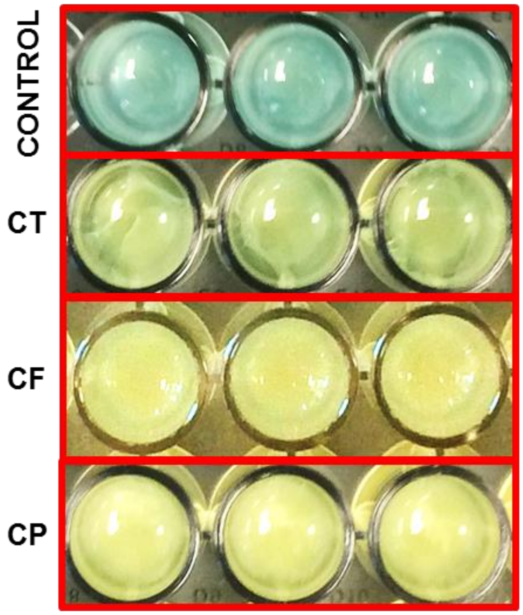

Figure-4: Inhibition of pyocyanin production of *P. aeruginosa* PAO1 by cephalosporins. Image of microtiter plate wells showing the inhibition of blue color (pyocyanin production) by *P. aeruginosa* PAO1 in the presence of CT (CT), CF (CF), and CP (CP).

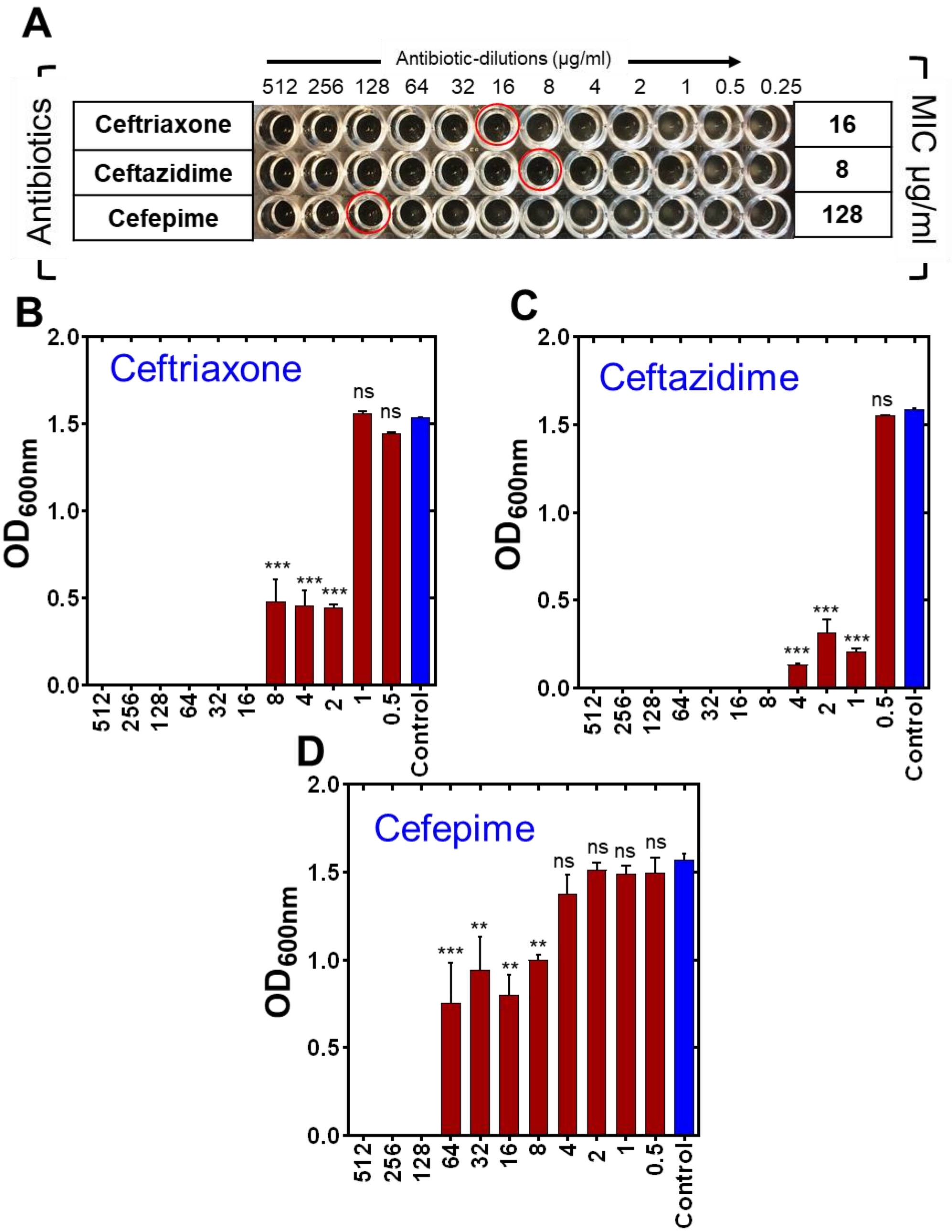

Supplementary figure 5: Minimum inhibitory concentration (MIC) determination of cephalosporins against *C. violaceum* CV026. Image of microtiter plate showing wells of PAO1 growth inhibition (A); red circle represents the well of visible growth inhibition. Graphical representation of OD $_{600nm}$ of *C. violaceum* CV026 with various concentrations of ceftriaxone (B), ceftazidime (C), and cefepime (D).

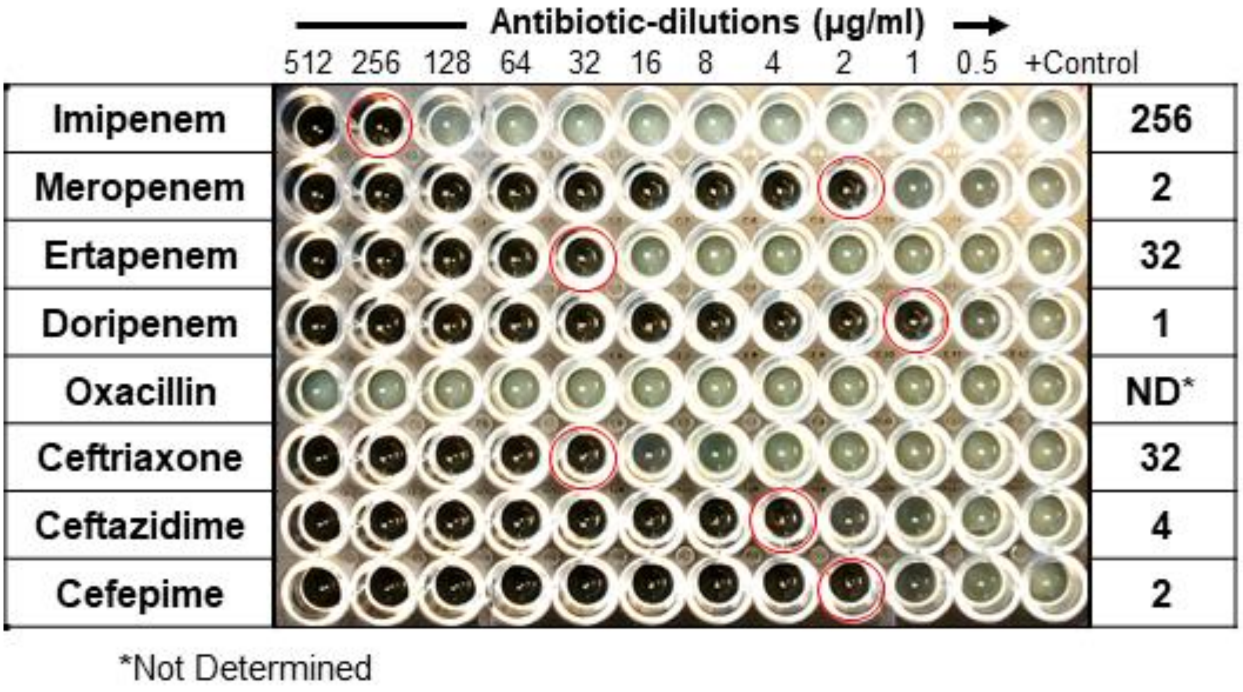

Supplementary figure-6: Minimum inhibitory concentration (MIC) determination of *Pseudomonas aeruginosa* PAO1 against cephalosporin antibiotics. Image of microtiter plate showing the growth inhibition. The names of the antibiotics are written on the left panel, and the MIC values are written on the right panel of the image.

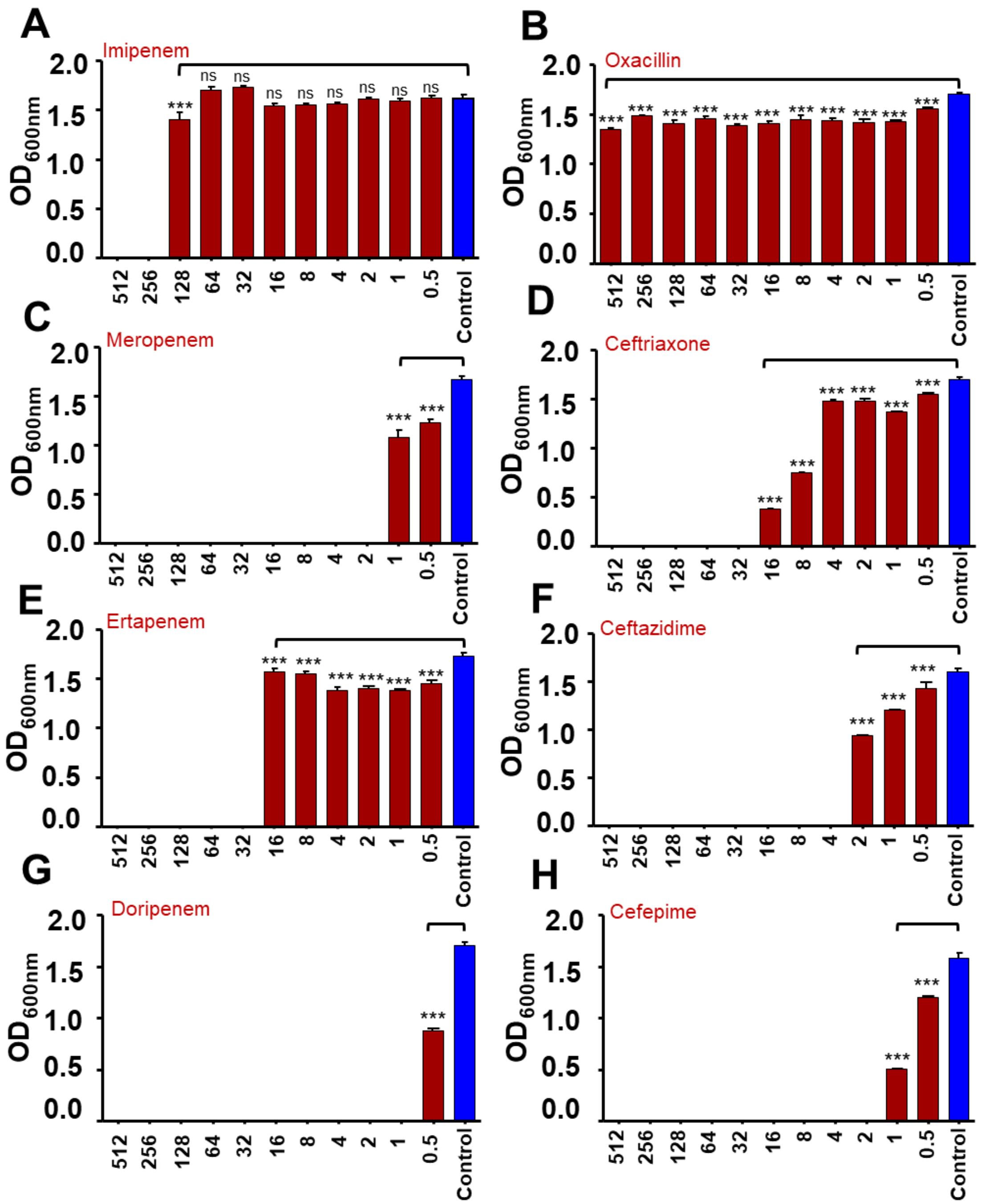

Supplementary figure-7: Minimum inhibitory concentration (MIC) determination of *Pseudomonas aeruginosa* PAO1 against Cephalosporins. Graphical representation of OD_{600nm} of *P. aeruginosa* PAO1 at different concentrations of cephalosporins (A-imipenem; B-oxacillin; C-Meropenem; D-ceftriaxone; E-ertapenem; F-ceftazidime; G-doripenem; and H-cefepime).

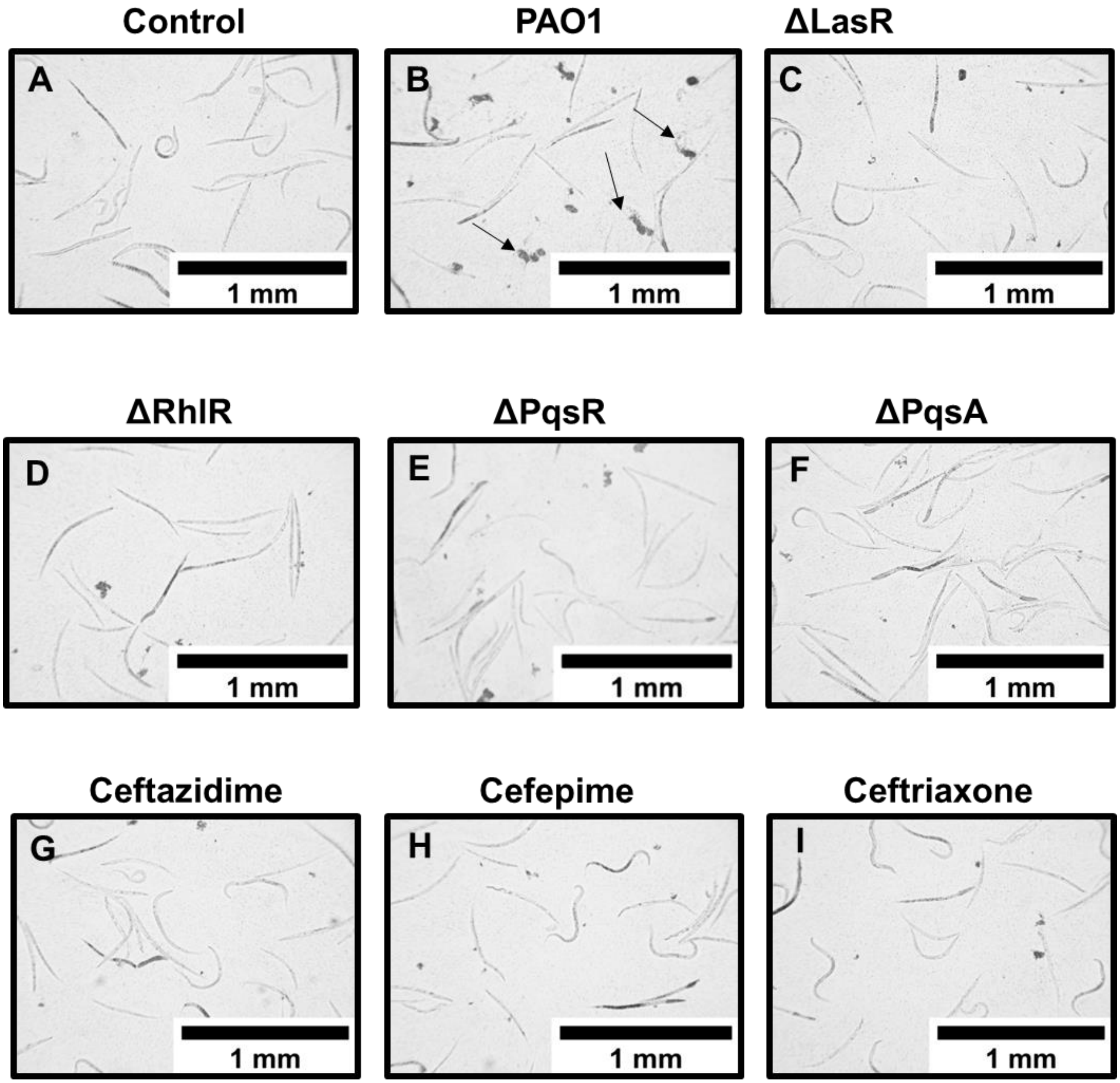

Supplementary figure-8: Antivirulence effect of cephalosporins on *C.elegans* survival. Image showing the appearance of *C. elegans* without any treatment (A); after exposing to the supernatant of PAO1 (B); after exposing to the supernatant of QS mutant strains; ΔLasR (C); ΔRhlR (D); ΔPqsR (E); ΔPqsA (F); and exposing to the supernatant of PAO1 grown in the presence of a sub-MICs of ceftazidime (G); cefepime (H), and ceftriaxone (I).

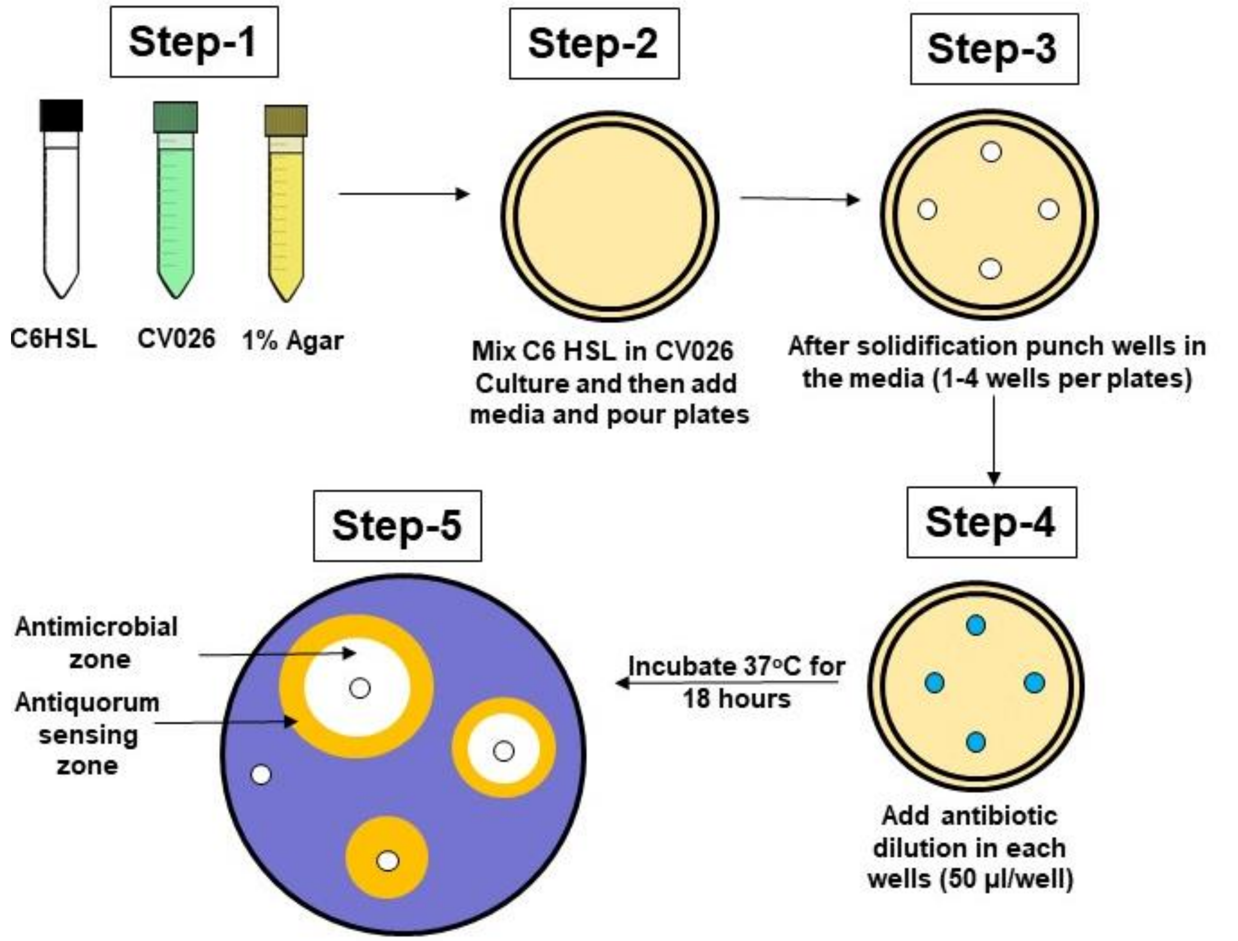

Supplementary figure-9: Schematic representation of agar well diffusion assay for the screening anti-QS activity of antibiotics using *Chromobacterium violaceum* CV026 as detector strain. Step-1: Preparation of signal molecule (C6-HSL), overnight *C. violaceum* CV026 culture, and 1% molten agar. **Step-2:** Making agar plates by mixing molten agar, *C. violaceum* CV026, and C6 HSL**. Step-3:** Punching wells using sterile pipette tips. **Step-4:** Filling wells with antibiotic dilutions. **Step-5:** Schematic representation of two distinct zones; antimicrobial zone and anti-QS zones (Interpretation of results of the assay: white zone indicates the antimicrobial zone and yellow zone with bacterial growth with no pigment production indicates anti-QS activity zone).

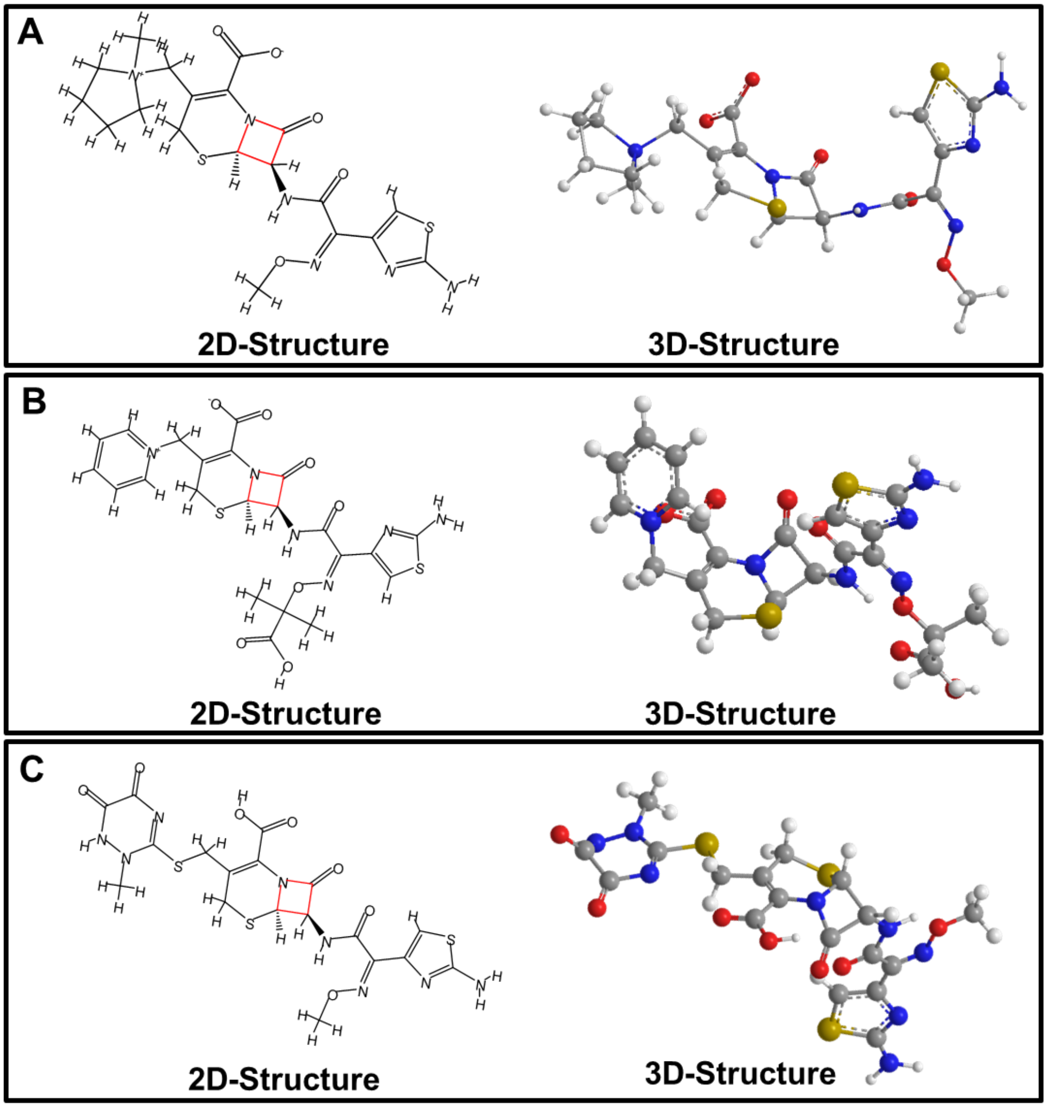

Supplementary figure-10: 2-D and 3-D chemical structure of cephalosporins; cefepime (A); ceftazidime (B); and ceftriaxone (C) (red square represents the beta-lactam ring of cephalosporin antibiotics. In the 3D structure of antibiotics nitrogen, hydrogen, carbon, oxygen, and sulfur are represented with blue, white, grey red, and light brown balls.

Effect of cephalosporins on the growth profile of *P. aeruginosa* PAO1. To estimate the anti-virulence and antibiofilm activities of the selected cephalosporin antibiotics, we studied the effect of sub-MICs of CP, CF, and CT on the growth of *P. aeruginosa* PAO1. Most of the cephalosporin antibiotics showed potent antimicrobial activity against PAO1 except oxacillin (no antimicrobial activity at 265 µg/mL). MIC values for imipenem, meropenem, doripenem, ertapenem, CT, CF and CP were found to be 256 µg/mL, 2 µg/mL, 32 µg/mL, 1 µg/mL, 32 µg/mL, 4 µg/mL, and 2 µg/mL (**Supplementary Fig 6**). Interestingly, the OD_{600nm} values at sub-MIC of each antibiotic were found to be significantly lower ($p<0.001$) than the control group (PAO1) (**Supplementary Fig 7A-7H**). This indicated that growth was influenced by antibiotics. Surprisingly the growth was also significantly affected in oxacillin antibiotics that showed no antimicrobial activity against PAO1 (512-0.5 µg/l) (**Supplementary Fig 6**). These results were encouraging and indicated that antibiotics were targeting alternate molecular pathways influencing the growth of *P. aeruginosa*. Further, we measured growth curves of PAO1 in the presence and the absence of sub-MIC (MIC, MIC/2, and MIC/4) of each antibiotic (**Fig 10A-2C**). We also compared the growth of PAO1 with QS mutant strains, as shown in **Fig 10D**.

For all three antibiotics, i.e., CP, CF, and CT, PAO1 growth was inhibited at the MIC and unaffected at zero and MIC/4 concentrations. However, at sub-MIC concentrations of MIC/2, growth became monophasic, deviating from the biphasic growth. The biphasic growth was absent for QS-mutant strains (**Fig 10D**). We fitted the growth curves with the equation (Liquori et al., 1981):

$$OD_{600} = \frac{a(1-e^{-t/t_1})}{1-e^{-t/t_1}+e^{-t/t_2}} + \frac{b(1-e^{-t/t_3})}{1-e^{-t/t_3}+e^{-t/t_4}} + c \qquad (1)$$

Equation (1) fitted (solid lines) data points (symbols) well (**Fig 10A-2D**). Typically, a monophasic growth described by a logistic equation is observed in a bacteria culture. There are three distinct phases: (1) a lag phase with very slow growth when the bacteria undergo preparatory steps for the next phase, (2) a log phase of exponential growth when the bacteria divide at a constant rate, (3) a stationary phase when the bacteria stop dividing due to unfavorable conditions such as nutrient scarcity. At the stationary phase, *P. aeruginosa* leverages QS pathways and responds to nutrient scarcity by producing virulence factors such as proteases (Cicmanec and Holder, 1979) and pyocyanin (Whooley and McLoughlin, 1982;Allen et al., 2005) to trigger a multipronged survival strategy. Proteases can break down large protein molecules available in the extracellular space to smaller components to be used as nutrients (Cicmanec and Holder, 1979), whereas pyocyanin can kill competitive bacteria or organisms (Baron and Rowe, 1981). As a result, a new phase of growth is facilitated by QS pathways resulting in the observed biphasic growth (**Fig 10A-2C**). This conclusion is supported by the absence of the second growth phase in all QS-mutant strains of PAO1 (**Fig 10D**). An analysis of the measured stationary phases for QS-mutant reveals that the ΔLasR mutant has a stationary phase with the smallest slope. In contrast, the ΔPqsA mutant has a stationary phase with the largest slope. **Fig 10D** suggests that the inhibition of QS by the four mutants follows the order: ΔLasR>ΔPqsR>ΔRhIR>ΔPqsA. This order agrees with the known hierarchical order of QS pathways in *P. aeruginosa* (Lee and Zhang, 2015b). The first phase of the growth curve also revealed an interesting observation. The QS-mutations did not affect the first phase of growth and matched well with the first phase of PAO1, suggesting that the first phase is not controlled by QS pathways. For cephalosporins, CP and CT showed the least and most effects respectively on the preparatory lag phase of the first monophasic growth. The MICs for CP, CF,

and CT against PAO1 were measured to be 2 µg/mL, 4 µg/mL, 32 µg/mL, respectively, i.e., CP is the most potent among the three against PAO1 (**Supplementary Fig 6**). Despite the low MIC, sub-MIC concentrations did not delay the start of the exponential log phase of PAO1 growth, suggesting that CP induced less environmental stress to PAO1, leading to a shorter preparatory lag phase. 1/2th MIC concentration was significantly affecting the growth of the cells and delaying the log phase, while 1/4th MIC did not affect the growth profile of the bacteria. Cells were effectively growing in the presence of these concentrations of antibiotics (**Fig 10A and Fig 2D**). Therefore, 1/4th MIC concentration of each antibiotic was selected to test anti-virulence and anti-biofilm activities against PAO1. These findings agree with previous reports that show these antibiotics alter gene expression induced mutations and alter growth profiles at sub-MICs leading to growth inhibition of bacteria (Shockman and Lampen, 1962;López and Blázquez, 2009).

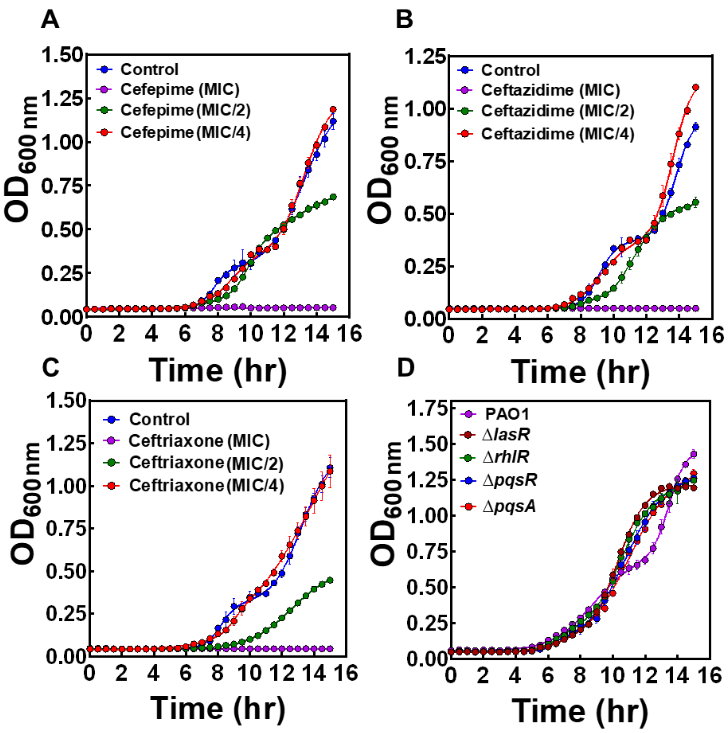

Supplementary figure-10: Effects of cephalosporins on the biphasic growth of *P. aeruginosa* PAO1. Growth curves of PAO1 at different concentrations (MIC, MIC/2, and MIC/4) of CP (A), CF (B), and CT (C), respectively. Growth curves of *P. aeruginosa* PAO1 and its isogenic QS-mutant strains (ΔLasR, ΔRhlR, ΔPqsA, and ΔPqsR) (D).

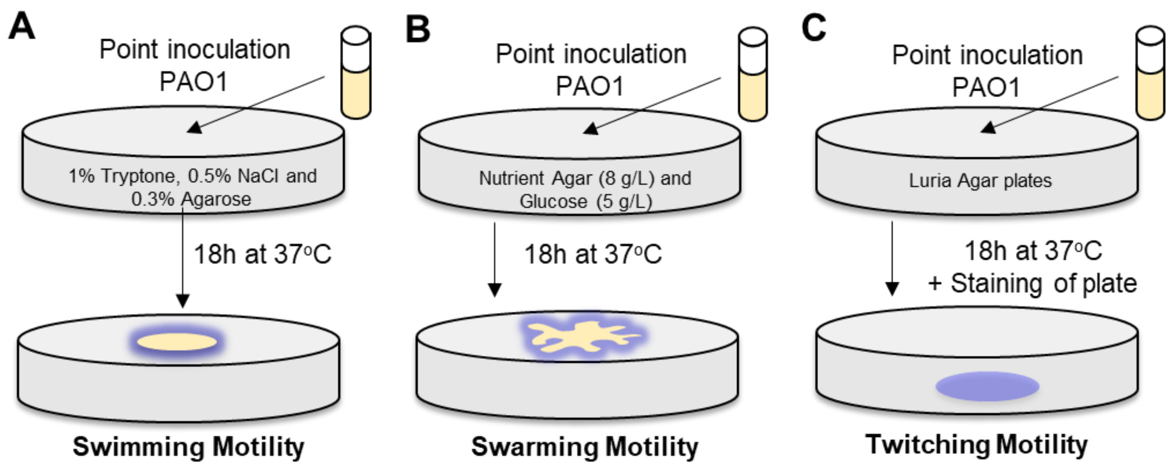

Supplementary figure-11: Schematic showing the method of motility inhibition testing. (A) swimming motility, (B) swarming motility, and (C) twitching motility.

Supplementary Table 1 The grid-box coordinates of respective quorum sensing receptors used for molecular docking experiments

S. No.	Protein	Grid-box coordinates	Value
1.	CviR	Center_x	30.862
		Center_y	35.116
		Center_z	18.001
		Size_x	30
		Size_y	42
		Size_z	34
2.	LasR	Center_x	9.714
		Center_y	2.032
		Center_z	20.29
		Size_x	36
		Size_y	30
		Size_z	28
3.	PqsR	Center_x	-52.182
		Center_y	3.466
		Center_z	10.195
		Size_x	22
		Size_y	22
		Size_z	22

Note: Exhaustiveness was set to 24 in each simulation.

Supplementary Table-2: Effect of cephalosporin treatment of the motility of *P. aeruginosa* PAO1 and QS mutant strains. Table showing the diameter of zone (mm) of swimming (A), swarming (B), and twitching (C) motility phenotypes for PAO1 and its isogenic QS mutant strains. Not significant (ns): $p > 0.05$, * $p \leq 0.05$, ** $p \leq 0.01$, *** $p \leq 0.001$, **** $p \leq 0.0001$.

A

Strains	PAO1	Δ*lasR*	Δ*rhlR*	Δ*pqsR*	Δ*pqsA*
Control	3.46 ± 0.05	3.13 ± 0.25 ns	3.00 ± 0.02 ns	3.03 ± 0.15 ns	3.33 ± 0.25 ns
Cefepime	1.03 ± 0.15 ***	0.53 ± 0.32 ns ***	0.60 ± 0.17 ***	0.63 ± 0.25 ***	0.70 ± 0.20 ***
Ceftriaxone	1.40 ± 0.17 ***	1.26 ± 0.40 ***	1.13 ± 0.23 ***	1.16 ± 0.05 ***	1.10 ± 0.01 ***
Ceftazidime	1.53 ± 0.15 ***	1.30 ± 0.43 ***	1.16 ± 0.15 ***	1.23 ± 0.05 ***	1.30 ± 0.01 ***

B

Strains	PAO1	Δ*lasR*	Δ*rhlR*	Δ*pqsR*	Δ*pqsA*
Control	3.43 ± 0.41	1.86 ± 0.32 *	1.07 ± 0.36 *	3.16 ± 0.028 ns	3.33 ± 0.32 ns
Cefepime	1.20 ± 0.10 ***	0.80 ± 0.10 ***	0.70 ± 0.10 ***	0.90 ± 0.30 ***	0.96 ± 0.35 ***
Ceftriaxone	2.03 ± 0.15 ***	1.53 ± 0.05 ***	1.50 ± 0.1 ***	1.86 ± 0.05 ***	1.86 ± 0.15 ***
Ceftazidime	2.26 ± 0.35 ***	1.46 ± 0.05 ***	1.70 ± 0.01 **	2.03 ± 0.15 ***	2.03 ± 0.32 ***

C

Strains	PAO1	Δ*lasR*	Δ*rhlR*	Δ*pqsR*	Δ*pqsA*
Control	3.20 ± 0.30	1.56 ± 0.20 *	1.70 ± 0.10 *	2.83 ± 0.11 ns	2.73 ± 0.32 ns
Cefepime	0.80 ± 0.10 ***	0.50 ± 0.10 ***	0.46 ± 0.05 ***	0.60 ± 0.10 ***	0.66 ± 0.05 ***
Ceftriaxone	1.16 ± 0.15 ***	0.56 ± 0.15 ***	0.56 ± 0.25 ***	1.03 ± 0.15 ***	0.96 ± 0.20 ***
Ceftazidime	3.13 ± 0.15 ns	2.13± 0.25 ns	2.33± 0.15 ns	2.86± 0.05 ns	2.83± 0.15 ns
